# Control of Somatosensory Cortical Processing by Thalamic Posterior Medial Nucleus: A New Role of Thalamus in Cortical Function

**DOI:** 10.1371/journal.pone.0148169

**Published:** 2016-01-28

**Authors:** Carlos Castejon, Natali Barros-Zulaica, Angel Nuñez

**Affiliations:** Departamento de Anatomía, Histología y Neurociencia, Facultad de Medicina, Universidad Autónoma de Madrid, Madrid, Spain; University of Sussex, UNITED KINGDOM

## Abstract

Current knowledge of thalamocortical interaction comes mainly from studying lemniscal thalamic systems. Less is known about paralemniscal thalamic nuclei function. In the vibrissae system, the posterior medial nucleus (POm) is the corresponding paralemniscal nucleus. POm neurons project to L1 and L5A of the primary somatosensory cortex (S1) in the rat brain. It is known that L1 modifies sensory-evoked responses through control of intracortical excitability suggesting that L1 exerts an influence on whisker responses. Therefore, thalamocortical pathways targeting L1 could modulate cortical firing. Here, using a combination of electrophysiology and pharmacology *in vivo*, we have sought to determine how POm influences cortical processing. In our experiments, single unit recordings performed in urethane-anesthetized rats showed that POm imposes precise control on the magnitude and duration of supra- and infragranular barrel cortex whisker responses. Our findings demonstrated that L1 inputs from POm imposed a time and intensity dependent regulation on cortical sensory processing. Moreover, we found that blocking L1 GABAergic inhibition or blocking P/Q-type Ca2+ channels in L1 prevents POm adjustment of whisker responses in the barrel cortex. Additionally, we found that POm was also controlling the sensory processing in S2 and this regulation was modulated by corticofugal activity from L5 in S1. Taken together, our data demonstrate the determinant role exerted by the POm in the adjustment of somatosensory cortical processing and in the regulation of cortical processing between S1 and S2. We propose that this adjustment could be a thalamocortical gain regulation mechanism also present in the processing of information between cortical areas.

## Introduction

Cortical functioning cannot be properly understood without taking into account the thalamic influence [1–9]. Knowledge of thalamocortical influence in sensory processing comes mainly from studying lemniscal core thalamic systems that project to granular layers of primary sensory cortices [3, 7, 10]; however, less is known about paralemniscal thalamic systems.

In the rodents, vibrissal information is conveyed to the somatosensory cortex via several parallel pathways [11–19]. In the lemniscal pathway, the ventral posterior medial nucleus of the thalamus (VPM) projects to L4, L5B and L6A in the primary somatosensory cortex (S1). In the extralemniscal pathway, the ventral tier of VPM projects mainly to L4 and L6 [67] in the secondary somatosensory cortex (S2). And in the paralemniscal pathway, the posterior medial nucleus (POm) projects to L1 and L5A in S1 and also to S2 [18–24]. It has been proposed that, whereas these ascending pathways appear to be parallel anatomically, they may not be functionally equivalent [39].

Thalamic VPM nucleus can be considered a “First order” relay station [5, 9], receiving sensory information from the principal trigeminal nucleus (PrV). POm nucleus is largely more complex to classify since it receives sensory information from the interpolar division of the spinal trigeminal nucleus (SpVi) and also from L5 of the somatosensory cortical areas [5, 15, 21, 25, 26]. There are several important differences between both nuclei: VPM is topographically well organized [19, 27–30]. In contrast, POm neuronal responses show poor spatial resolution [1, 15, 27, 31] with receptive fields composed of multiple vibrissae [12]. Recordings from both nuclei revealed different adaptation process to repetitive stimuli [1, 32, 33]. Offset latencies remained constant in POm neurons across the different stimulation frequencies [1]. In agreement with those findings, other studies found that onset and offset latencies of SPVi paralemniscal neuronal responses were not affected by deflecting the vibrissae at different frequencies [32, 34]. These properties of paralemniscal neurons render them poorly suited for coding specific stimulus content features. It has been proposed that signals conveyed by the lemniscal pathway involve high-resolution encoding of contact and texture information relayed from the vibrissae [17, 35]. The role of POm and the paralemniscal system in sensory processing is less clear. It is known that paralemniscal system processes temporal features of tactile stimuli [1, 34], and is involved in nociceptive transmission [32, 36–38]. Also, it has been suggested that POm neurons represent (temporal- to rate-code transformation by thalamocortical loops) the temporal frequency of whisker movements by latency and spike count [1, 34] and that the POm is involved in temporal processing related to sensory-motor control of whisker movement [17, 34, 35]. Other authors have reported that whisking in air, without vibrissae contacts, fails to evoke significant activity in POm neurons [32]. Actually, the nature and function of the messages that POm thalamic nucleus transfers to the cortex are still under debate.

It has been proposed that the role of the paralemniscal projection is to provide modulatory inputs to barrel cortex [39]. However, the possible mechanisms by which these projections could regulate the cortex are unknown.

Here, we have sought to determine POm influences in cortical processing by single-unit recordings in somatosensory cortex of urethane-anesthetized rats. Our findings demonstrate that POm modulates magnitude and duration of S1 cortical responses to sensory input. We found that GABAergic inhibitory transmission in L1 is implicated in the regulation of cortical excitability and sensory response magnitude and duration. Our results are consistent with a previous work that described L1 inhibitory influence on whisker responses [40]. Accordingly, we demonstrate that POm exerts its control of cortical sensory responses mainly through L1.

Additionally, it has been suggested that ‘Higher order’ thalamic nuclei play a key role in corticocortical communication [41, 42]. In S1, L5 corticofugal neurons send the processed information to the POm and to various subcortical regions [9, 15, 25, 26, 43]. Recently, both anatomical and physiological findings have shown that ascending inputs from the brainstem and descending inputs from L5 converge on single thalamocortical neurons in POm [25]. Both individual pathways interact functionally in a time-dependent manner [25]. From here, POm neuron projections also target other cortical areas including the primary motor cortex (M1) and higher-order somatosensory cortical regions [18, 21]. Furthermore, it is well described the reciprocal connections between these areas. These connections are likely to play a crucial role in sensory-motor integration and sensory learning. However, both the function of that transthalamic pathway and the nature of the messages that are relayed through the POm from one cortical area to another remain unclear.

In this study, we propose that cortical sensory response modulation by POm could be also present in the processing of information between somatosensory cortical areas. We performed a complementary set of experiments to test this hypothesis and found that POm is also controlling the sensory processing in S2 and this regulation is modulated by corticofugal activity from L5 in S1 [25]. In sum, our findings demonstrate the determinant role exerted by the POm in the adjustment of barrel cortex sensory processing and in the regulation of cortical processing between somatosensory cortical areas.

## Materials and Methods

### Animal procedures and electrophysiology

All animal procedures were approved by the Ethical Committee of the Universidad Autonoma de Madrid, in accordance with European Community Council Directive 2010/63/UE. Rats were group housed with a 12-h light/dark cycle and had free access to food and water. Every effort was made to minimize the number and suffering of the animals used. Experiments were performed on 98 (36 males and 62 females) urethane-anesthetized (1.6 g/kg i.p.) adult Sprague Dawley rats weighing 200–250 g. Animals were placed in a Kopf stereotaxic frame in which surgical procedures and recordings were performed. The animals breathed freely. The body temperature was maintained at 37°C; the end-tidal CO_2_ and heart rate were monitorized. Local anaesthetic (Lidocaine 1%) was applied to all skin incisions. The level of anesthesia was monitored and kept constant (absence of whisker movements and pinch withdrawal reflex) using supplemental doses of urethane. The skull was exposed and then openings were made to allow electrode penetrations to different neuronal stations in the cortex, thalamus and brainstem. Tungsten microelectrodes (2–5 MΩ) were driven using a microdrive system. Extracellular recordings were made of putative excitatory neurons in the interpolar division of the ipsilateral spinal trigeminal complex (SpVi; AP 11.5–14; L 2.5–3.5, D 8.5–9.5; in mm from Bregma; [44], contralateral posterior medial nucleus (POm; AP 2.5–4.5, L 2–2.5, D 5–6.5) of the thalamus and contralateral vibrissal region of the primary (S1; AP 0.5–4, L 5–7) and secondary (S2; AP 0–3.7; L 7–7.5) somatosensory cortices. In S1, barrel cortical neurons were recorded in supragranular (D: 200–600 μm) or infragranular (D: 900–1500 μm) layers. In S2, neurons were recorded along the cortical depth (D: 400–1300 μm). To estimate the depths of recorded neurons, we used the micromanipulator axial depth readings.

### Sensory stimulation

Controlled whisker deflections were performed by brief air puffs (20–200 ms) applied to one whisker (deflected in caudal direction) at 0.5 Hz using a pneumatic pressure pump (Picospritzer) that delivers an air pulse through a 1 mm inner diameter polyethylene tube (1.2–2 kg/cm^2^) avoiding skin stimulation. We choose this precise stimulus to assure the effect of our protocols and to avoid complex, likely nonphysiological responses. Vibrissae were cut 9 mm from the skin in order to allow a controlled mechanical stimulation of single vibrissae and to evoke reproducible responses. Details on train duration, pulse duration and number of consecutive deflections applied are provided in figures. We determined receptive field size of single units by deflecting individual vibrissae with a hand-held probe and monitoring the audio conversion of the amplified activity signal.

### Electrical stimulation

Electrical microstimulation was carried out with single square pulses (0.5 ms, 5–80 μA; S88 Grass Stimulator). We applied these pulses at 0.5 Hz to avoid possible adaptation phenomena. Electrical stimulation (E-stimulation) was applied in POm, VPM, L5 or L1 in S1 cortex, using 120 μm diameter stainless steel bipolar electrodes. The E-stimulation parameters were digitally controlled by Spike2 software (Cambridge Electronic Design, Cambridge, UK) and transmitted to the current source via a digital-to-analog converter built in to the CED Power 1401 data acquisition unit (Cambridge Electronic Design). We tried to establish the minimal, but effective, stimulation parameters for detecting changes in cortical neural responses and to avoid possible antidromic activity [45] in order to study only orthodromic effects. Stimulation within the current range used in our study (<80 μA) is estimated to activate cells within a maximal radius of 0.5 mm [46]. At the end of each E-stimulation experiment we applied a train of 20 pulses (0.5 ms; same intensity) at high frequency (100 Hz) to check for antidromic activity. We did not find evoked spikes having the ability to follow this high frequency E-stimulation. Thus, none of the cortical recorded neurons were antidromically activated by thalamic E-stimulation at the intensities used. None of the E-stimulation parameters used here induced subtle motor effects, whisking or facial twitching.

We identify the placement of the electrodes on histological sections or according to their response pattern. Only the data from cases in which the electrode tip was unambiguously well localized inside the corresponding thalamic nucleus or cortical layer were quantitatively analyzed.

### Pharmacological study

The following drugs were used: Muscimol (5-(aminomethyl)-isoxazol-3-ol; selective agonist for γ-aminobutyric acid receptor-A (GABA_A_) receptors; 1 mM), Picrotoxin (PTX; prototypic antagonist of GABA_A_ receptors; 1mM) and Cav2.1 (P/Q- type) voltage-gated calcium channels blocker ω-agatoxin-IVa (AGA; 0.1 μM). Drugs were injected through a cannula connected to a Hamilton syringe (1 μl). The syringe was driven using a microdrive system to inject the drug solution into the cortical surface or into the thalamic nucleus (AP 3.3 mm, L 2.5 mm to the Bregma for POm, or L 3.2 mm for VPM and D 4.8–6.8 mm from the surface of the brain; [44]). The piston of the syringe was moved manually at a slow speed (infusion speed 0.3 μl/min). A volume of 0.1 to 0.3 μl of muscimol was infused unilaterally into the corresponding thalamic nuclei. PTX or AGA was applied to the surface of the cortex (1 μl). Given the potential of GABA_A_ receptors antagonists, PTX in our experiments, to induce seizures (e.g. [47–49]), all rats were carefully monitored for indicators of seizures after infusions. None of the PTX injections elicited tremor, motor convulsions or more subtle seizure effects such as jaw or facial twitching.

### Histology

Upon completion of the experiments, animals were deeply anesthetized with sodium-pentobarbital (50 mg/kg) and then perfused transcardially with saline followed by formalin (4% in saline). Subsequently, 50 μm thick sections were prepared for Nissl staining for verification of cannula placement and to locate the stimulation and recording electrode tracks. Placements of the lesions were determined using a light microscope and mapped onto coronal sections of a rat brain stereotaxic atlas [44].

### Data acquisition and analysis

Raw signal was filtered (0.3–3 kHz band pass), amplified via an AC preamplifier (DAM80; World Precision Instruments, Sarasota, USA), and fed into a computer (sampled at 10 kHz) with the temporal references of the stimuli for off-line analysis. Single-unit activity was extracted with the aid of commercial software Spike2 (Cambridge Electronic Design, Cambridge, UK) for spike waveform identification and analysis. Furthermore, we also supervise waveforms to confirm that units were well isolated. The sorted spikes were stored at a 1-ms resolution and isolated single-units were analyzed and quantified. We defined response magnitude as the total number of spikes per stimulus occurring between response onset and offset from the peristimulus time histogram (PSTH, bin width 1 ms). Response onset was defined as the first of three consecutive bins displaying significant activity (three times higher than the mean spontaneous activity) after stimulus and response offset as the last bin of the last three consecutive bins displaying significant activity. Response duration was defined as the time elapsed from the onset to offset responses. The baseline firing rate was calculated from mean firing within a 10 s window before the first stimulus (air puff). In all figures, raster plots represent each spike as a dot for sample neuron. Spikes were aligned on stimulus presentation (Time 0 ms). In some figures, PSTHs and rasters are shown from multi-units recordings just to clarify the effects.

Statistical analysis was performed using GraphPad Prism 5 software (San Diego, CA, USA). For all experiments, data analysis was based on single unit responses. For normally distributed data (Shapiro-Wilk normality test), comparisons of activities of single units in different conditions were performed by using paired two-tailed t test, where P<0.05 was considered significant. Data are presented as means ± SEM. Non-normally distributed data were compared with Wilcoxon-matched pairs test (as indicated in the text).

## Results

Our experiments were designed to study thalamic POm influence in somatosensory cortical processing. First, we studied and characterized the firing pattern of POm responses to whisker deflections. After that, to test whether POm activity modulates cortical tactile processing, we investigated whisker response changes in barrel cortex by electrically stimulating the POm immediately before whisker stimulus or by muscimol-induced inactivation of the POm. Finally, we pharmacologically blocked GABAergic inhibitory transmission in L1 to understand the contribution of this layer in POm regulation of cortical processing.

Additionally, to determine the possible role exerted by the POm in the adjustment of somatosensory cortical processing between S1 and S2, we performed a complementary set of experiments investigating whisker response changes in S2 by electrically stimulating S1 and by muscimol-induced inactivation of the POm.

### POm responses lasted the duration of the stimulus

**Fig 1:**
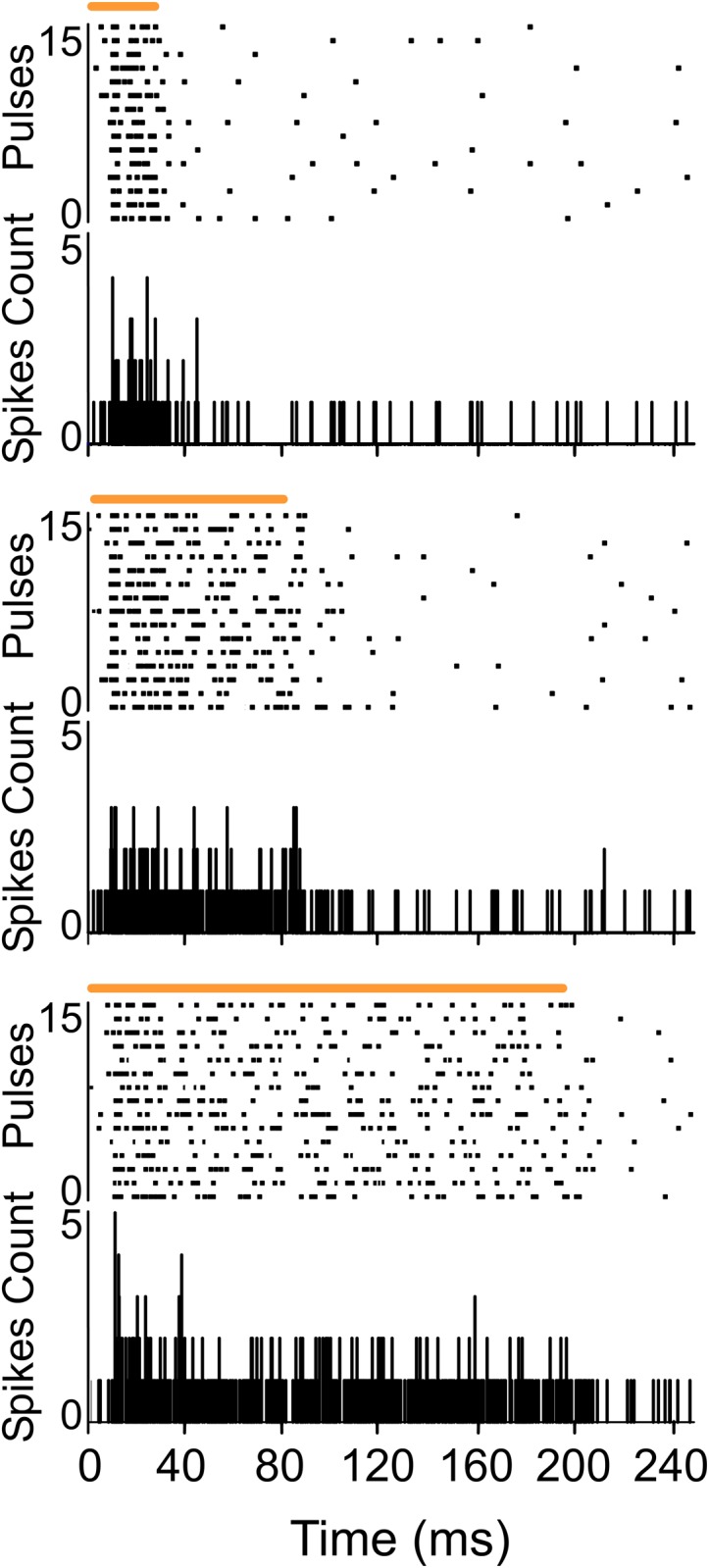
POm responses lasted the duration of the whisker stimulus. Raster plots and PSTHs showing sustained multiunit POm responses evoked by different stimulus duration (top: 20 ms, middle: 80 ms and bottom: 200 ms). Air puff duration is indicated by horizontal orange lines.

Performing experiments in 10 rats, we firstly characterized the firing pattern of POm neurons delivering air-puffs of different durations (20–200 ms) to one whisker, avoiding skin stimulation. We found multivibrissae receptive fields (mean receptive field size: 6.1±2.5 vibrissae; range: 3–12; n = 118) at all POm recording sites. Our recordings from POm revealed a sustained response along stimulus presence, as shown by the raster of spikes in response to 0.5 Hz periodic vibrissae deflections (Fig 1). Specifically, 72% of the recorded neurons exhibited this response pattern (85 of 118). We also found that 83% of the recorded neurons in SpVi exhibited the same pattern (85 of 102; data not shown). These findings demonstrated the presence of this sustained response pattern along the paralemniscal pathway.

### POm activity modulates sensory cortical processing

To examine the influence of POm nucleus on infra- and supragranular neurons in barrel cortex, we compared whisker responses in several experimental conditions increasing or decreasing POm activity.

#### POm E-stimulation evokes orthodromic spikes in infra- and supragranular layers of barrel cortex

**Fig 2:**
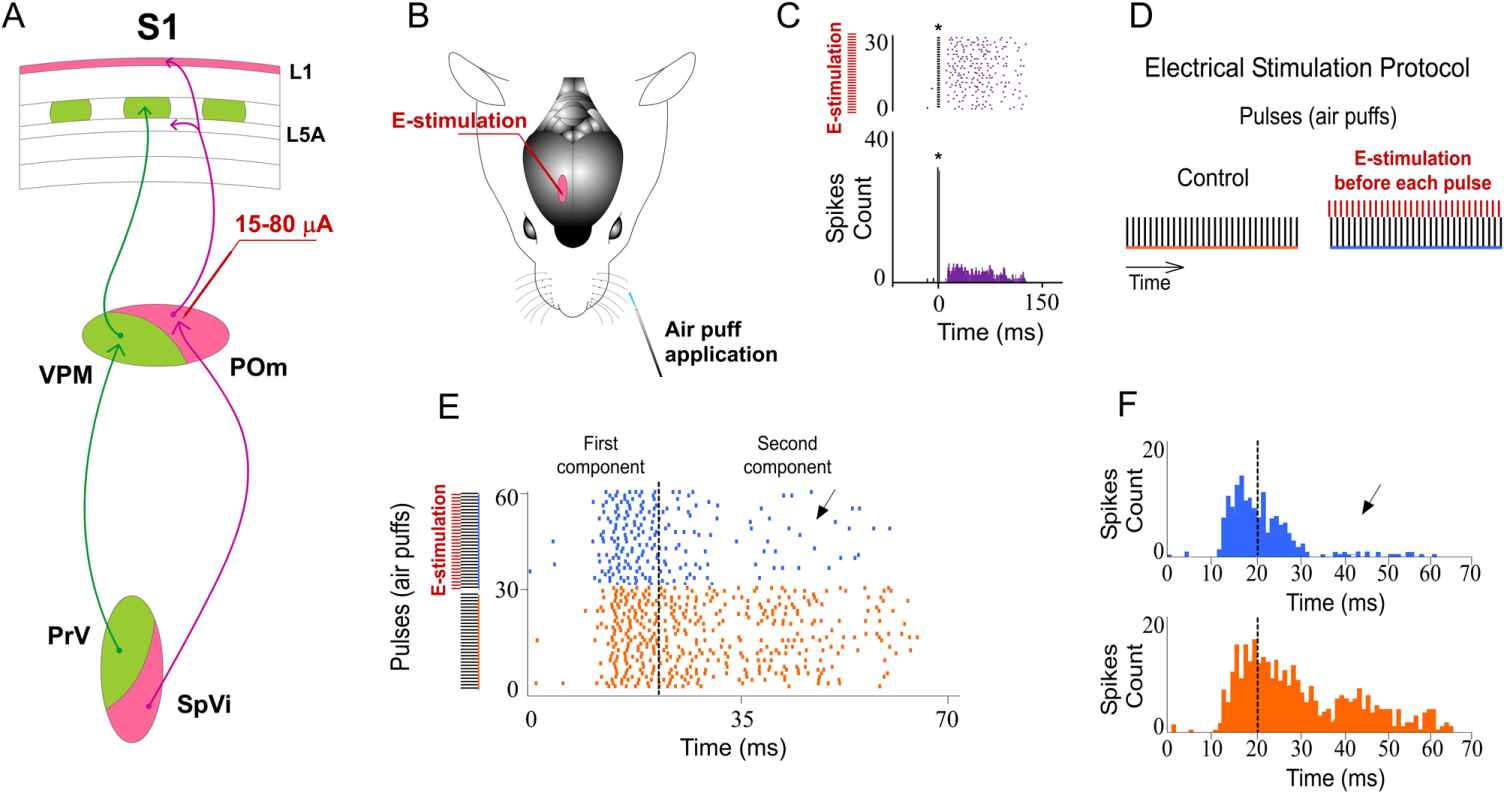
Increasing POm activity by POm E-stimulation just before sensory stimulus modulates whisker cortical responses. (A) Schematic diagram summarizing the main components of the lemniscal (green) and paralemniscal (pink) thalamocortical circuitry to barrel cortex. (B) Schematic diagram indicating the experimental protocol used in our study. (C) In agreement with recent studies suggesting that POm projections make excitatory synapses with barrel cortex pyramidal cells [20, 39, 43, 73], POm E-stimulation alone (single pulse of 0.5 ms; 15–80 μA) elicited orthodromic spikes in infra- and supragranular layers of barrel cortex. An example of evoked orthodromic spikes in the barrel cortex infragranular layer by POm E-stimulation is shown. * indicates stimulation artifacts. (D) Experimental protocol. The ‘Electrical Stimulation Protocol’ consisted of two blocks of 30 pulses (air puff 20 ms) delivered to the principal whisker at 0.5 Hz. We stimulated electrically the POm, VPM, L1 or L5 in S1 50–1000 ms before each pulse in the second block (blue) applied 60 s after the first block (orange). (E, F) POm E-stimulation 500 ms before whisker stimulus reduced sensory responses in infra- and supragranular layers of S1. Raster plots (E) and PSTHs (F) are shown for a sample multiunit infragranular response. Vertical dashed lines separate response components. POm E-stimulation shortened responses and reduced spikes mainly in the second response component (arrows). Spikes are aligned on sensory stimulus (air puff) presentation (Time 0 ms). POm E-stimulation was applied 500 ms before air puffs (31 to 60 pulses; red bars).

We investigated whisker response changes in barrel cortex by POm electrical stimulation (E-stimulation) immediately before whisker stimulus (air puff) application in 15 rats (Fig 2). We restricted our recordings to infra- and supragranular layers of barrel cortex (see Discussion). Cortical neurons were silent or displayed a low firing rate in spontaneous conditions (0.89±0.1 spikes/s in infragranular layers, n = 69; 0.69±0.1 in supragranular layer, n = 51). Whisker deflections caused short-latency spikes in infra- and supragranular layers of barrel cortex. All neurons displayed a contralateral response to whisker displacements. Spike shape and firing pattern (low spontaneous firing rate and reduced tactile response to the deflection) provide strong support to the notion that recordings were obtained from pyramidal cells, as has been previously reported [50–54].

First, we stimulated electrically the POm (single pulse of 0.5 ms; 15–80 μA) alone. POm E-stimulation elicited spikes (for example see Fig 2C) in infra- and supragranular layers of barrel cortex. In infragranular layers the latencies of these spikes varied in the range of 5–50 ms (mean latency: 23.67±0.9 ms; n = 69). In supragranular layers in the range of 5–50 ms (mean latency: 16.30±0.5 ms; n = 51). These findings are in agreement with recent studies suggesting that POm projections make excitatory synapses with barrel cortex pyramidal cells [20, 39, 43].

Also, in all cases, we checked for potential rebound excitation (potential delayed spikes >150 ms in infra- or >50 ms in supragranular layers after E-stimulation offset). However, after POm E-stimulation we did not find rebound excitation even at maximal intensity used in our experiments (80 μA;).

Anatomically POm receives corticothalamic inputs from infragranular layers, thus, infragranular activity elicited by thalamic POm E-stimulation could also result from antidromic activation of corticothalamic axons. This would induce cortical responses characterized by minimal response variability and failure to show neural response fatigue [55, 56]. In contrast, orthodromic stimulation would activate cortical sites through neural pathways, characterized by substantial response timing variability and decremental cortical responses with repeated electrical stimulation pulses. At the end of each E-stimulation experiment we applied a train of 20 pulses (0.5 ms; same intensity) at high frequency (100 Hz) to check for antidromic activity. We did not find evoked spikes having the ability to follow this high frequency E-stimulation. None of the cortical recorded neurons were antidromically activated by thalamic E-stimulation at the intensities used. Thus, the results obtained in our experiments were due to orthodromic cortical activation from thalamic inputs.

#### Increasing POm activity by E-stimulation modulates sensory response in barrel cortex

To examine the effects of POm E-stimulation on infra- and supragranular neurons, we compared cortical sensory responses before and during POm E-stimulation (500 ms before each vibrissae stimulus; Fig 2). We applied the E-stimulation protocol defined by two blocks of 30 pulses (air puff 20 ms duration) delivered to one whisker at 0.5 Hz. In the second block, we stimulated electrically the POm just before each sensory stimulus (Fig 2D). Quantitative measures of neural responses were examined to determine how paralemniscal thalamic E-stimulation affected cortical responses to vibrissae deflections. We found that POm E-stimulation was accompanied by a marked change in cortical sensory responses in a layer specific manner. Following POm E-stimulation just before whisker stimulus, cortical sensory response magnitude and duration significantly decreased. These effects were demonstrated both by the rasters and by the peristimulus time histograms (PSTHs; Fig 2F). Results were consistent across all animals (n = 15).

Taken into account that POm projections target specifically L5A, we performed a preliminary analysis of single-units from different deeps. POm E-stimulation induced similar response decrease in both superficial (900–1200 μm) and deep (1200–1500 μm) infragranular units (-27%; n = 27; P<0.001 and -33%; n = 31; P<0.001, respectively). A total of 82% of superficial infragranular neurons (27 of 33) and 86% of deep infragranular neurons (31 of 36) decreased their sensory responses correlated with POm E-stimulation. Therefore, we combined all these single units across different depths into a single neuronal population termed infragranular layer. In this cortical layer, POm E-stimulation before each vibrissae stimulus induced a mean response decrease from 2.08±0.1 spikes/stimulus in control condition (before the application of the POm E-stimulation) to 1.48±0.1 spikes/stimulus during POm E-stimulation condition (-29%; n = 80; P< 0.001). A total of 89% of infragranular neurons (80 of 90) displayed changes in responses correlated with POm E-stimulation. The latency of the vibrissae response onset did not change while offset latencies significantly decreased during POm E-stimulation. Onset tactile responses had on average 13.20±0.12 ms latency in control and 13.09±0.10 ms after POm E-stimulation (-1%; n = 80; P = 0.41). Offset tactile responses decreased on average from 59.64±0.55 in control condition to 46.14±0.32 ms during POm E-stimulation (-23%; n = 80; P< 0.001).

In supragranular layers, POm E-stimulation applied before each whisker stimulus induced a mean response decrease from 1.95±0.1 spikes/stimulus in control condition to 1.33±0.1 spikes/stimulus in POm E-stimulation condition (-32%; n = 67; P < 0.001). A total of 90% neurons (67 of 74) displayed changes correlated with POm E-stimulation. Onset tactile responses had on average 14.92±0.22 ms latency and 14.14±0.18 ms after POm E-stimulation (-4%; n = 67; P = 0.06). Offset tactile responses had on average 41.64±0.6 ms latency and was reduced to 32.81±0.71 ms after POm E-stimulation (-21%; n = 67; P<0.001).

In both layers, POm E-stimulation before whisker stimulus resulted in decreased spike count. However, this reduction was not homogeneous along the sensory response (Fig 2E and 2F). Previous reports from our laboratory have described two different components of tactile responses and the relevant implication of N-methyl-D-aspartate (NMDA) receptors manly in the late component of the response [57, 58]. Accordingly, here we divided each PSTH in two components: the first (from onset to 20 ms) and the second (from 20 ms to offset) components. We found important differences between these components. In infragranular layers, the first component of the PSTH did not decrease (from 1.02±0.1 to 0.97±0.1 spikes/stimulus; -5%; n = 80; P = 0.44). However, spikes were suppressed abruptly in the second component of the response by POm E-stimulation (from 1.06±0.1 to 0.50±0.1 spikes/stimulus; -52%; n = 80; P<0.001). In supragranular layers, the first component decreased from 1.15±0.1 to 0.94±0.1 (-19%; n = 67; P< 0.001) and from 0.80±0.1 to 0.40±0.1 (-50%; n = 67; P<0.001) in the second component. These findings demonstrate the important differences between both components.

**Fig 3:**
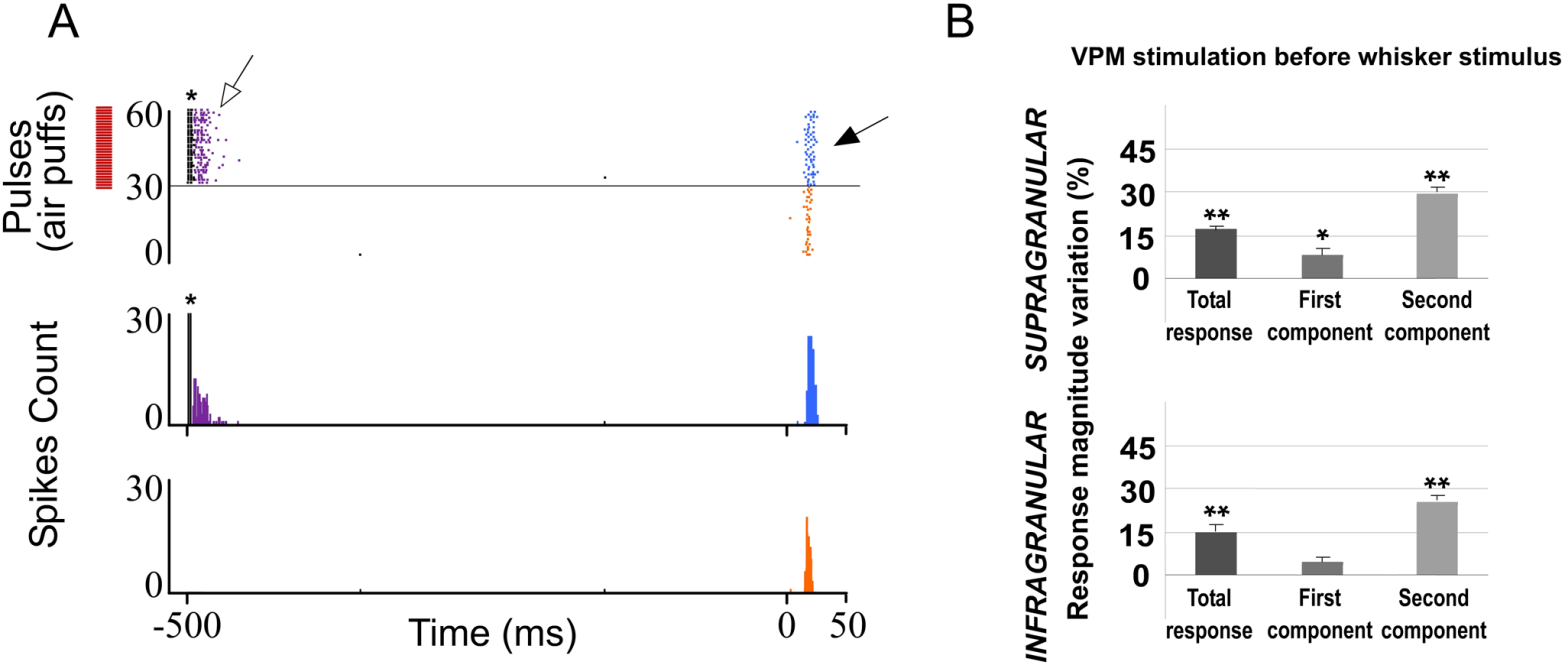
VPM E-stimulation just before whisker stimulus enhances sensory responses in barrel cortex. (A) Raster plots and PSTHs are shown for a sample supragranular neuron. VPM E-stimulation was applied 500 ms before pulses 31 to 60 (red bars). In contrast to POm E-stimulation, spikes mainly in the second component of the response were strongly increased by VPM E-stimulation (filled arrow). We did not find delayed rebound excitation occurring 30 ms after VPM E-stimulation within the current range used in our study (<80 μA). VPM E-stimulation evoked cortical spikes (open arrow). * indicates E-stimulation artifacts. (B) Change (%) in mean sensory response magnitude by VPM E-stimulation 500 ms before stimulus. Total response was increased in both layers by VPM E-stimulation. First component of infragranular responses was not significantly affected. Spikes in the second component were strongly increased in both layers.

As a control for specificity of the POm E-stimulation site, we also stimulated electrically (single pulse of 15–80 μA, 0.5 ms) the VPM in 9 rats. We found that VPM E-stimulation alone elicited short latencies spikes in infra- and supragranular layers of barrel cortex. In infragranular layers the latencies of these spikes varied in the range of 4–38 ms (mean latency: 13.42±0.5 ms; n = 38) and in supragranular layers in the range of 4–30 ms (mean latency: 12.37±0.4 ms; n = 50). We also applied high frequency VPM E-stimulation (a train of 20 pulses at 100 Hz). Cortical spikes decreased with increasing pulse number consistent with orthodromic stimulation. Also, we test the possibility of rebound excitation. We did not find delayed rebound excitation occurring 38 ms after VPM E-stimulation (for example see Fig 3A) within the current range used in our study (<80 μA). However, applying VPM E-stimulation with a higher intensity (>130 μA) we found rebound activity in same tested cases (data not shown).

After that, we compared cortical sensory responses before and after VPM E-stimulation (500 ms before each stimulus). Cortical responses increased their magnitude in both layers (quantified in Fig 3B; P<0.001in both layers). This effect was more prevalent in the second component of the response. In infragranular layers, we found an increased number of spikes from 0.96±0.1 to 1.01±0.1 spikes/stimulus (5%; n = 38; P = 0.031) in the first component. Spikes in the second component of the response were also increased by VPM E-stimulation (from 1.09±0.2 to 1.36±0.2 spikes/stimulus; 25%; n = 38; P<0.001; Fig 3). Similarly, the number of spikes in the first component increased from 1.13±0.1 to 1.21±0.1 (7%; n = 50; P<0.001) and from 0.69±0.1 to 0.89±0.1 (29%; n = 50; P<0.001; Fig 3) in the second component of supragranular layer neurons. A total of 73% of infragranular layer neurons (38 of 52) and 76% of supragranular neurons (50 of 66) displayed increments in responses correlated with VPM E-stimulation.

These findings suggest significant differences between POm and VPM thalamic nuclei. Our results showed that VPM E-stimulation alone elicited shorter latencies orthodromic spikes in infra- and supragranular layers of barrel cortex than POm E-stimulation. VPM orthodromic spikes varied in the range of 4–38 ms in infra- and of 4–30 ms in supragranular layers. However, POm E-stimulation (same intensity) elicited evoked-spikes lasting up to 150 ms in infra- and up to 50 ms in supragranular layers. These results are in agreement with other studies showing that evoked bursts of EPSCs in neocortical neurons triggered by VPM neurons had faster decay times than those from POm neurons [20].

Moreover, in contrast to VPM E-stimulation, following POm E-stimulation just before whisker stimulus, cortical responses magnitude and duration significantly decreased. These opposite results from VPM or POm E-stimulation on whisker cortical responses suggested a different functional role of these thalamic nuclei in somatosensory processing.

#### POm inactivation enhances whisker response magnitude and duration in barrel cortex

**Fig 4:**
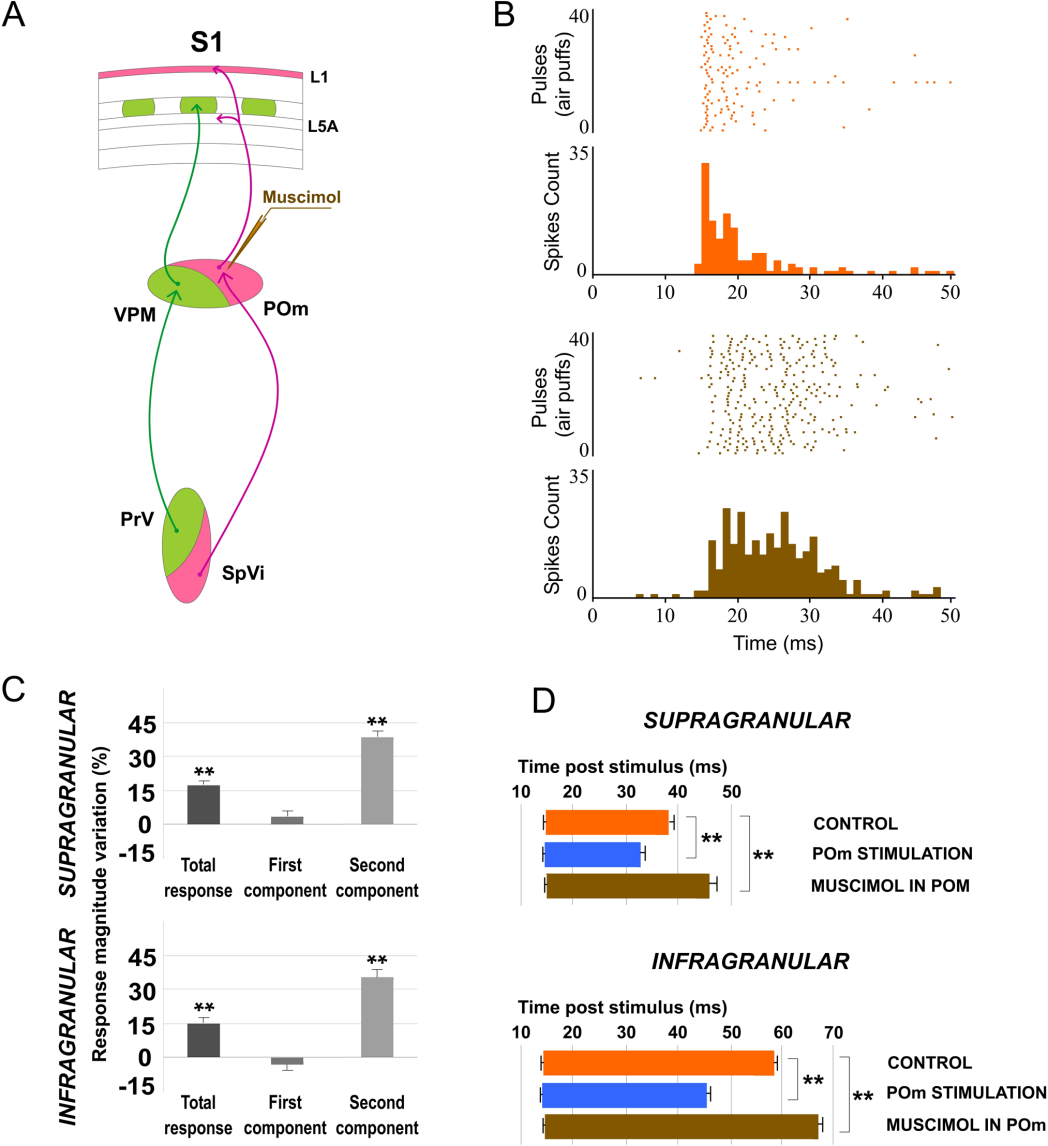
Muscimol-induced inactivation of the POm. (A) Schematic diagram indicating the experimental manipulation of the paralemniscal (pink) thalamocortical circuitry to barrel cortex. (B) POm inactivation enhanced responses in S1 mainly in the second component. Raster plots and PSTHs are shown for a sample supragranular neuron before (top) and after (bottom) POm inactivation. Also the pattern of spikes in the response was changed after POm inactivation suggesting that POm imposes a precise control of cortical responses. (C) Percentage change in mean response magnitude when POm was inactivated with muscimol. Spikes were strongly enhanced in the second component of the response. (D) Mean onset and offset latencies and response duration in Control (orange), in POm E-stimulation (blue) and in POm inactivation condition (brown). Response duration decreased with POm E-stimulation and increased in POm inactivation condition. We did not find differences in onset latencies but offset latencies changed significantly. Horizontal bars represent response duration.

To further understand the POm implication in cortical sensory processing, we pharmacologically inactivated POm neurons by infusing a small volume (0.1–0.3 μl; 1 mM) of the GABA_A_ receptor agonist muscimol in 16 rats. Surprisingly, inactivating POm enhanced sensory responses in infra- and supragranular layers within a few minutes (<5 min) of the injection (Fig 4). We found enhanced tactile responses in 37 out of 51 neurons (67%) and 51 of 59 neurons (86%) in infra- and supragranular layer, respectively (measured at 15 min after injection). The evoked spikes in response to whisker stimulation were enhanced from 1.96±0.3 spikes/stimulus to 2.26±0.3 spikes/stimulus (15%; n = 37; P<0.001) in infragranular layers and from 1.86±0.2 spikes/stimulus to 2.16±0.3 spikes/stimulus (16%; n = 51; P<0.001) in supragranular layers (Fig 4C). The response facilitation was evident in the second response component (Fig 4C). In infragranular layers, the first component was not affected (from 1.04±0.2 to 1.03±0.2 spikes/stimulus; -2%; n = 37; P = 0.4). In contrast, spikes in the second component of the response were increased abruptly by POm inactivation (from 0.92 ± 0.1 to 1.23 ± 0.2 spikes/stimulus; 34%; n = 37; P<0.001). In supragranular layers, the first component was also not affected (from 1.13±0.2 to 1.17±0.2 spikes/stimulus; 3%; n = 51; P = 0.61) while the second component increased from 0.73±0.1 to 1.01±0.1 spikes/stimulus (37%; n = 51; P<0.001). The response onset latency was not significantly modified under muscimol application in POm (Fig 4D). However, the response offset latency was increased in infra- (12%; n = 37; P<0.001) and supragranular layers (22%; n = 51; P<0.001; Fig 4D).

Also, spontaneous activity was increased from 0.94±0.2 to 1.23±0.3 spikes/s (31%; n = 51; P<0.001) in infragranular neurons and from 0.70±0.2 to 0.95±0.2 spikes (35%; n = 37; P<0.001) in supragranular neurons. These results suggest that POm activity modulates cortical excitability of the barrel cortex.

**Fig 5:**
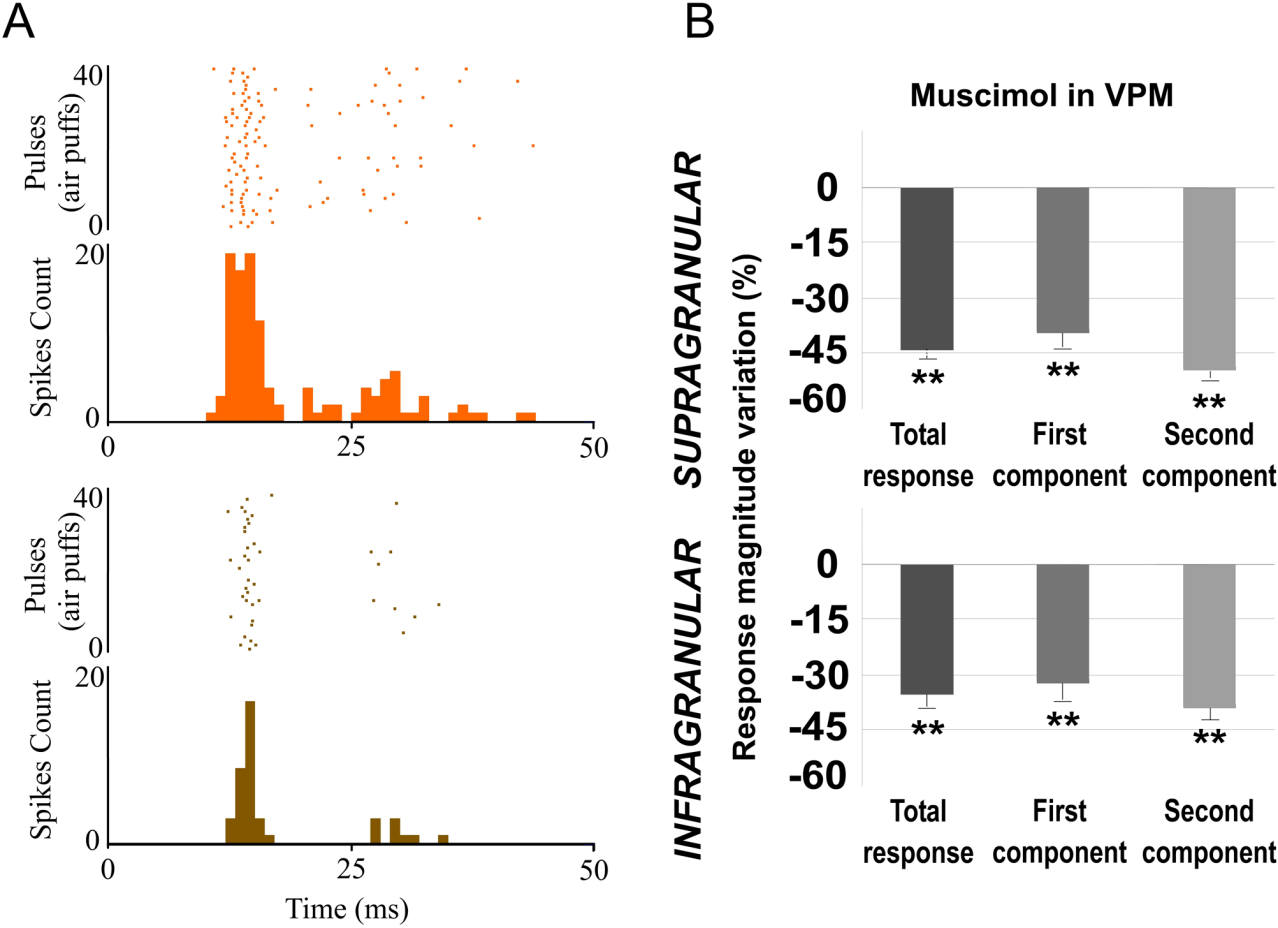
Muscimol-induced inactivation of the VPM. Inactivating VPM decreased responses in infra- and supragranular layers of S1. (A) Raster plots and PSTHs are shown for a supragranular sample neuron before (orange) and after (brown) VPM inactivation. (B) Mean response magnitude change (%) evoked by VPM inactivation. In both layers, spikes of sensory responses were strongly abolished after VPM inactivation by muscimol.

To determine if this effect was specific of POm nucleus, we pharmacologically inactivated VPM neurons with muscimol (0.1–0.3 μl; 1 mM) in 9 rats. The magnitude of cortical responses diminished within a few minutes (<5 min) after the injection (Fig 5). A total of 82% of infragranular layer neurons (27 of 33) and 94% of supragranular layer neurons (29 of 31) displayed significant reduction in responses correlated with VPM inactivation. The evoked spikes in response to whisker sensory stimulation were reduced from 1.97±0.3 to 1.29±0.2 spikes/stimulus (-34%, P<0.001; n = 27) in infragranular layers and from 1.72±0.3 to 0.97±0.2 spikes/stimulus (-44%, P<0.001; n = 29) in supragranular layers (Fig 5B). Our results are in agreement with other studies showing that VPM lesions abolish cortical responses evoked by whisker stimulation [59].

These findings suggest more differences between POm and VPM thalamic nuclei. In contrast to VPM inactivation, following POm inactivation cortical response magnitude significantly increased. In both layers, the first component was not affected by POm inactivation. However, spikes in both components of cortical sensory responses were strongly abolished after VPM inactivation. Again, these opposite results from VPM or POm inactivation on whisker cortical responses suggest a different functional role of these thalamic nuclei in somatosensory processing.

### POm regulation on cortical sensory processing is time and intensity-dependent

**Fig 6:**
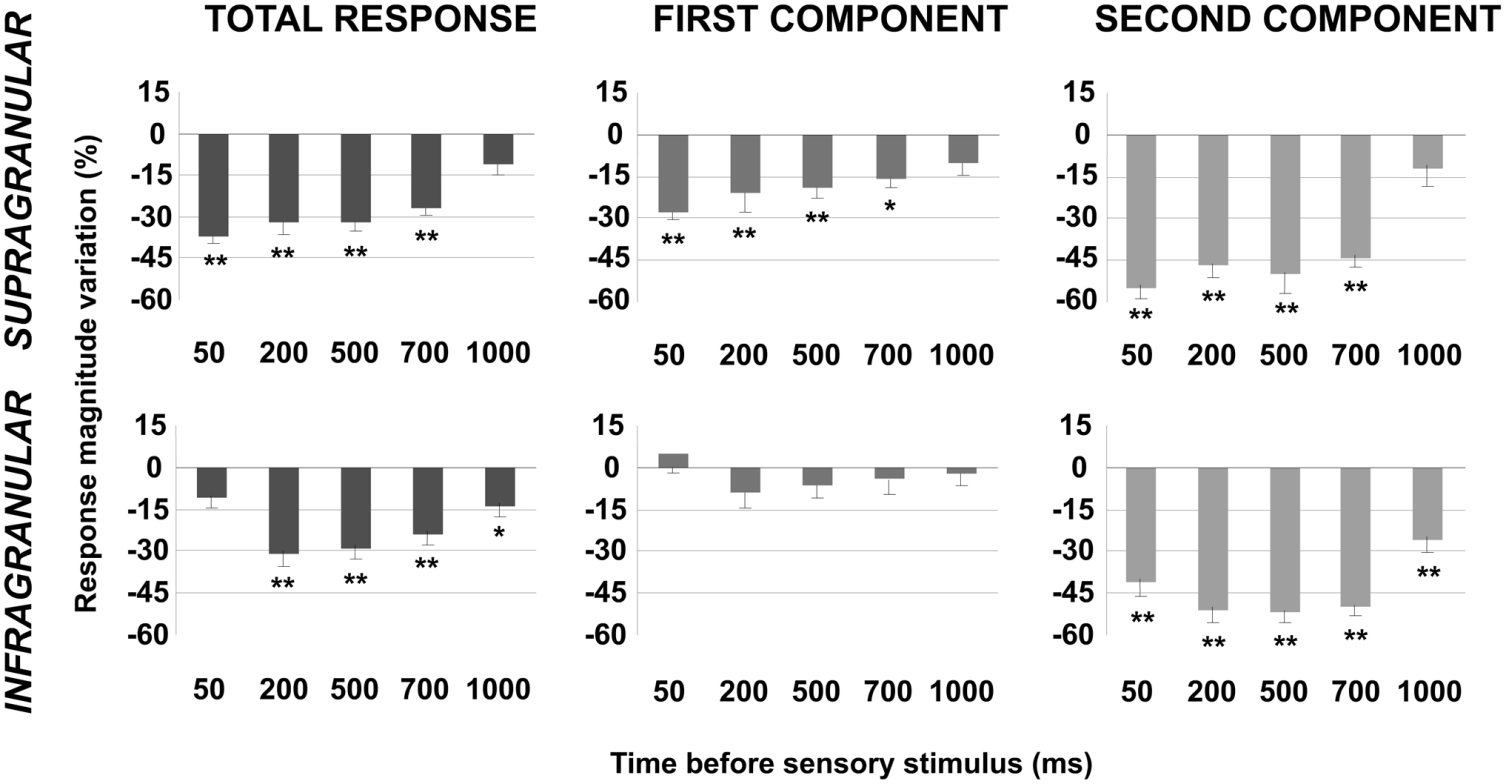
The effect of POm E-stimulation is time-dependent. This figure shows the change in mean sensory response magnitude by different POm E-stimulation intervals (50–1000 ms) before whisker stimulus. Supragranular total response was significantly reduced by POm E-stimulation at intervals ranged from 50 to 700 ms but not at 1000 ms. Total response of infragranular neurons was reduced at intervals from 200 to 1000 ms but not at 50 ms. In infragranular layers, the first response component (from onset to 20 ms) was not significantly affected at any time interval. In contrast, in supragranular layers, spikes in the first component were reduced at intervals <1000 ms. Spike reduction by POm E-stimulation was more prevalent in the second component of the responses in both layers (from 20 ms to offset). In supragranular neurons, spikes in the second component were decreased significantly at several time intervals from 50 to 700 ms before stimulus. The most powerful effect was found at 50 ms. Spikes in the second component of infragranular neurons were reduced significantly at time intervals from 50 to 1000 ms before sensory stimulus. In infragranular layer, the numbers of single units analyzed in each interval are: n = 38 (50 ms), n = 40 (200 ms), n = 80 (500 ms), n = 55 (700 ms) and n = 40 (1000 ms). In supragranular layer, n = 35 (50 ms), n = 33 (200 ms), n = 67 (500 ms), n = 45 (700 ms) and n = 32 (1000 ms). In all figures: * P<0.05; ** P<0.01.

To further understand these effects we investigated sensory response changes according to the interval between POm E-stimulation and sensory stimulus. We found that response magnitude and duration of cortical neurons changed by POm E-stimulation intervals before sensory stimulus. The results are summarized and quantified in Fig 6. This figure also demonstrates the important differences between both cortical layers. In infragranular layers, the first component was not significantly affected at any time interval (50–1000 ms). However, spikes in the first component were strongly reduced at all intervals in supragranular layers. In addition, we found a significant reduction of spikes in the second response component in both infra- and supragranular layers. Moreover, in supragranular layers, we did not find significant response changes at longer intervals than 700 ms. In contrast, we found a significant reduction of spikes even at 1000 ms in infragranular layer. These findings implicate different dynamics between both layers, especially on the first response component.

**Fig 7:**
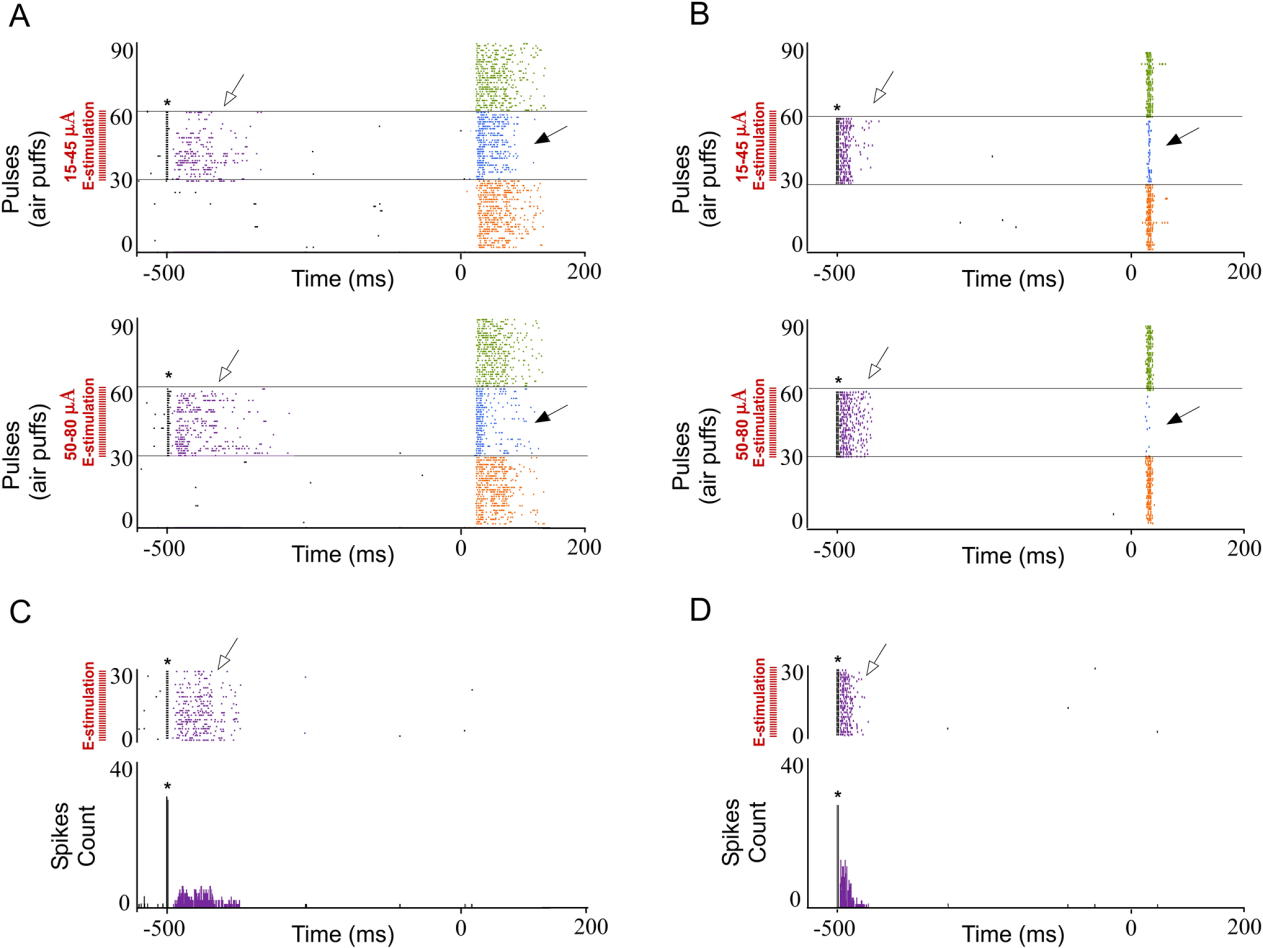
The effect of POm E-stimulation is intensity-dependent. Response duration and magnitude of cortical neurons decreased with increasing E-stimulation intensity. Raster plots and PSTHs are shown for a sample infragranular (A) and supragranular (B) responses after 15–45 μA (top) and 50–80 μA (bottom). Increasing POm E-stimulation intensity shortened responses more strongly, reducing spikes in the second response component (filled arrows). Control condition before (orange) and after (green) POm E-stimulation condition (blue) are shown. POm E-stimulation was applied 500 ms before pulses 31 to 60. Whisker stimulus presentation was applied at Time 0 ms. POm E-stimulation applied alone elicited orthodromic (open arrows) but not rebound activity in infra- (C) and supragranular layers (D). * indicates E-stimulation artifacts.

**Fig 8:**
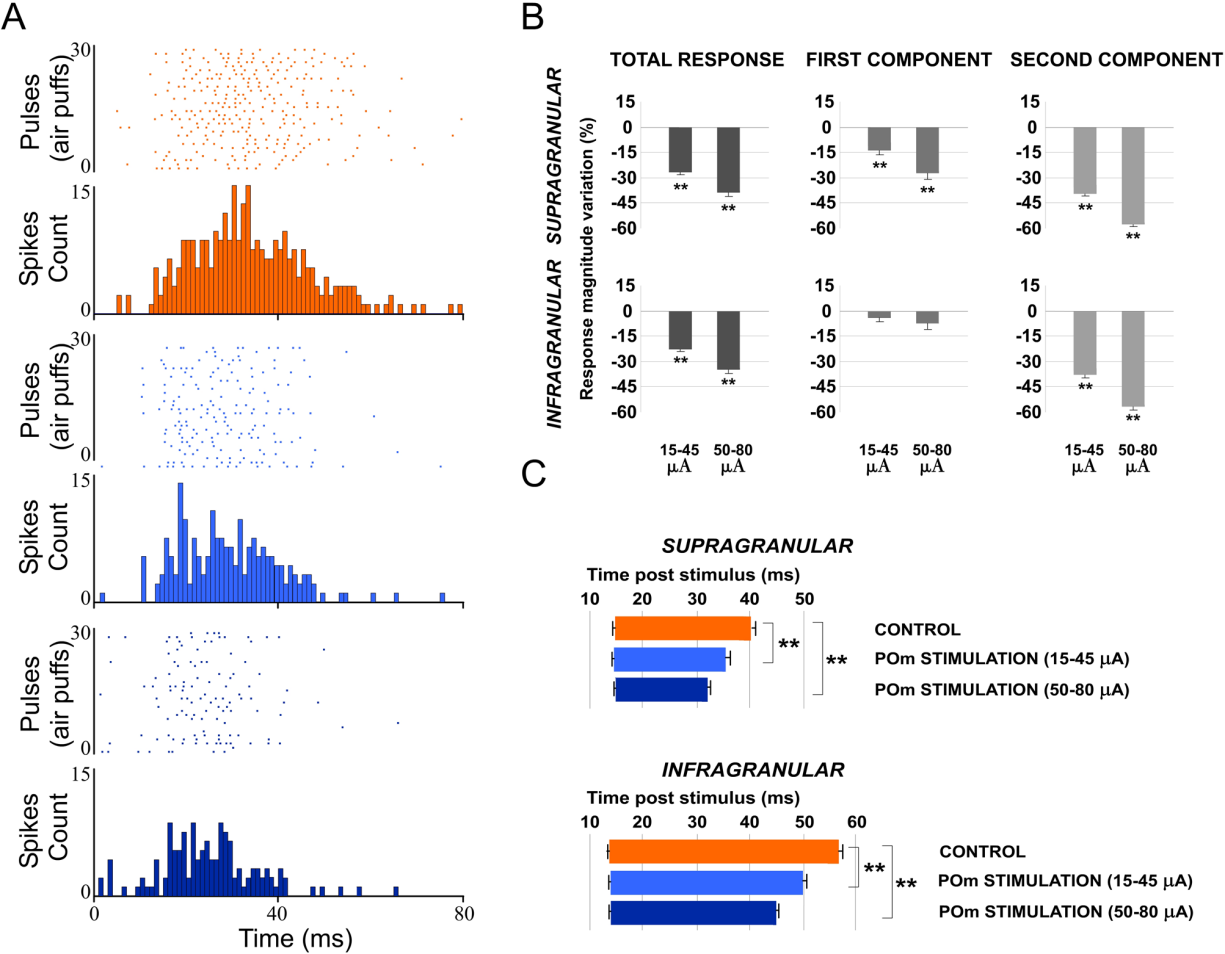
Increasing POm E-stimulation intensity enhances the reduction of second component spikes, shortening the duration of the sensory response. (A) Raster plots and PSTHs are shown for a supragranular whisker response change after increasing POm E-stimulation intensity before sensory stimulus. Control response (orange), 15–45μA POm E-stimulation (blue) and 50–80 μA POm E-stimulation (dark blue). (B) Response magnitude variation (%) with different POm E-stimulation intensities before sensory stimulus. (C) Increasing POm E-stimulation intensity before stimulus shortened the responses offset latencies. Horizontal bars represent response duration. In infragranular layers, the numbers of single units analyzed in B and C are: n = 57. In supragranular, n = 53.

We also found that response duration and magnitude of cortical neurons decreased with increasing E-stimulation intensity (Fig 7), indicating that POm E-stimulation effects are also intensity-dependent. Reduction in whisker response magnitude and duration by POm E-stimulation at two current intensity ranges (15–45μA and 50–80 μA) are quantified in Fig 8. We found that increasing POm E-stimulation intensity mainly reduced second component spikes, shortening the duration of sensory responses.

### POm exerts its control of cortical sensory responses mainly through L1

Recent studies suggest that POm projections make excitatory synapses with barrel cortex pyramidal cells [20, 39, 43]. Accordingly, we have showed above that POm E-stimulation alone elicited excitatory orthodromic spikes in infra- and supragranular layers of barrel cortex. However, our results also showed that POm E-stimulation just before sensory stimulus reduced magnitude and duration of cortical whisker responses. Moreover, POm inactivation by muscimol caused an enhancement of both sensory cortical responses and spontaneous cortical activity in the barrel cortex. How can these intriguing effects be explained? It is well described that blocking activity in L1 increases whisker-evoked responses [40], suggesting that L1 exerts an inhibitory influence on whisker responses. Since L1 receives strong inputs from POm [18, 19, 21, 23, 28], it is then possible that POm exerts its control of cortical sensory responses through L1. To test this hypothesis, we perform the following experiments.

#### Blocking inhibitory transmission in L1 enhances whisker response in barrel cortex

**Fig 9:**
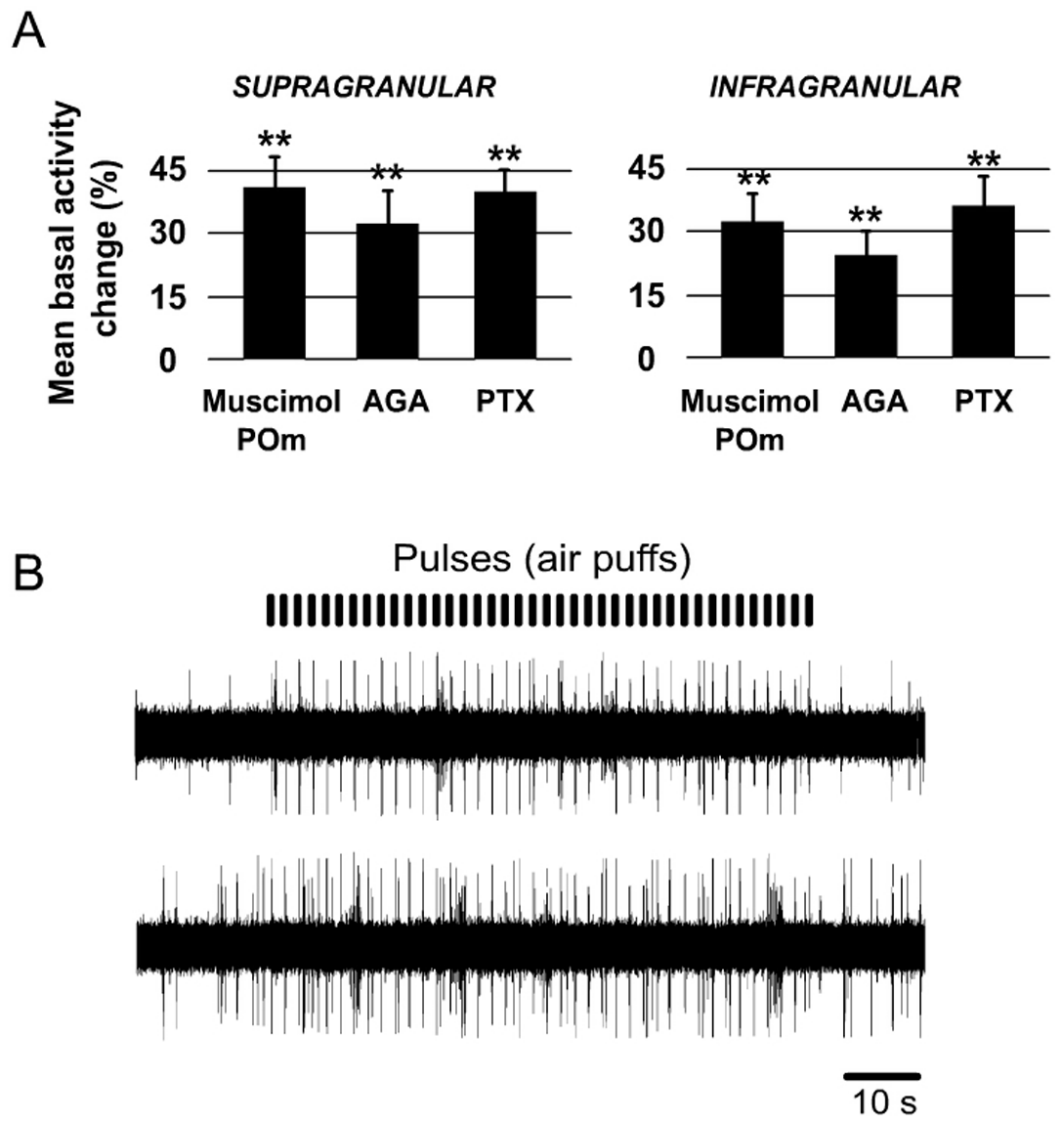
Cortical spontaneous activity changes in different tested conditions. (A) Mean basal activity change (%) in different conditions (muscimol in POm; AGA and PTX in cortical surface). In all conditions cortical basal activity in S1 was significantly increased after drugs applications. The baseline firing rate was calculated from mean firing within a 10 s window before the first pulse (air puff). (B) Muscimol-induced inactivation of the POm enhanced sensory responses in infra- and supragranular layers and increased cortical spontaneous activity. Raw cortical extracelular recordings are shown before (top) and after (bottom) muscimol application. These recordings show the enhancement of cortical sensory responses to whisker deflections (pulses). Cortical spontaneous activity was also increased after muscimol-induced inactivation of the POm.

It is known that L1 inputs generate direct, rapid excitatory postsynaptic potentials in L1 interneurons [60, 85]. Accordingly, in the barrel cortex, whisker-evoked sensory information is rapidly relayed to L1 neurons, which, in turn, act to powerfully inhibit whisker-evoked responses [40, 60]. Since L1 is composed of more than 90% of GABAergic neurons [61, 62], to further understand the contribution of L1 in POm regulation of cortical sensory processing, we pharmacologically blocked GABAergic inhibitory transmission in L1 in 12 rats. Picrotoxin (PTX; antagonist of GABA_A_ receptors; 1 mM) application (1 μl) to the cortical surface was accompanied by a marked change in cortical sensory responses in infra- and supragranular layers. Spontaneous activity rates were significantly affected, as is depicted in Fig 9. The baseline firing rate was increased from 0.88±0.3 to 1.20±0.3 spikes/s (36%; n = 22; P<0.001; Wilcoxon-matched pairs test) in infragranular layer and from 0.59±0.2 to 0.79±0.2 spikes/s (38%; n = 21; P<0.001; Wilcoxon-matched pairs test) in supragranular layer.

It is known that Cav2.1 (P/Q- type) voltage-gated calcium channels are expressed on parvalbumin (PV) interneuron axon terminals and mediate GABA release from fast spiking interneurons to pyramidal cells [63–65]. To study in more detail the inhibitory implication in POm control of cortical processing, we applied P/Q- type voltage-gated calcium channels blocker ω-agatoxin-IVa (0.1 μM) to the cortical surface (1 μl) in 10 rats. We found that cortical sensory response magnitude and duration significantly increased 15 min after injection. A total of 88% of infragranular layer neurons (23 of 26) and 91% of supragranular neurons (21 of 23) displayed increments in sensory responses after blocking P/Q-type calcium channels in superficial cortex. Spontaneous activity rates were also significantly affected (Fig 9). The baseline firing rate was increased from 0.97±0.3 to 1.18±0.3 spikes/s (22%; n = 23; P<0.001; Wilcoxon-matched pairs test) in infragranular layer and from 0.66±0.2 to 0.86 ± 0.2 spikes/s (31%; n = 22; P<0.001; Wilcoxon-matched pairs test) in supragranular layer.

**Fig 10:**
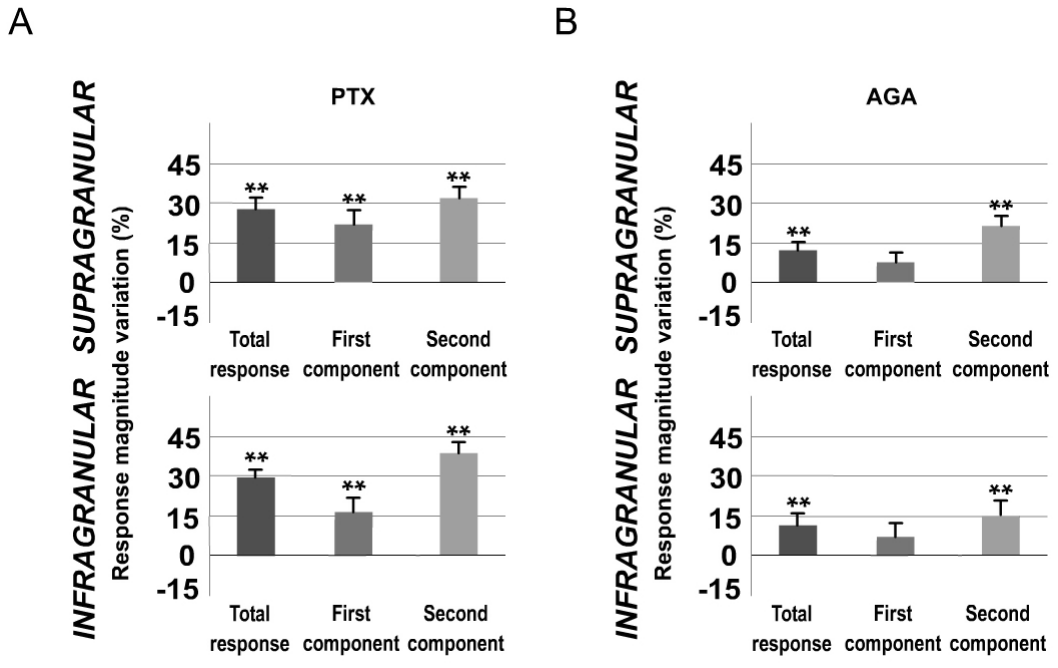
Blocking GABAergic inhibitory transmission in L1 enhances whisker responses. Change (%) in mean sensory response magnitude by PTX (A) or AGA (B) application in L1. Blocking GABAergic inhibitory transmission in L1 by PTX increased significantly whisker response magnitude in infra- (bottom row) and supragranular layers (top row) more strongly in the second component of the response. AGA application enhanced sensory responses in infra- and supragranular neurons. However, in both layers, AGA did not induce significant effects in the first component. * P<0.005; ** P<0.001.

A total of 92% of infragranular layer neurons (22 of 24) and 81% of supragranular neurons (21 of 26) displayed increments in sensory responses after blocking GABAergic inhibitory transmission in L1. Cortical response magnitude (Fig 10A) and duration were significantly increased 15 min after PTX application. Again, this effect was more prevalent in the second component of the response. In infragranular layers, we found an increased number of spikes from 0.84±0.2 to 0.98±0.2 spikes/stimulus (16%; n = 22; P<0.001; Wilcoxon-matched pairs test) in the first component. Spikes in the second component of the response were increased from 1.12±0.2 to 1.55±0.3 spikes/stimulus (38%; n = 22; P<0.001; Wilcoxon-matched pairs test; Fig 10A). In supragranular layers, we found an increased number of spikes from 0.97±0.2 to 1.19±0.2 (22%; n = 21; P<0.001; Wilcoxon-matched pairs test) in the first component and from 0.91±0.1 to 1.20±0.2 (32%; n = 21; P<0.001; Wilcoxon-matched pairs test; Fig 10A) in the second component.

In infragranular layers the latency of the response onset did not change (from 14.17±0.33 to 14.38±0.32 ms; 1%; n = 22; P = 0.37; Wilcoxon-matched pairs test) while offset latency increased from 55.46±0.67 to 69.29±1.54 ms (25%; n = 22; P<0.001; Wilcoxon-matched pairs test;). In supragranular layers the onset latency was not modified (from 14.35±0.36 to 14.64±0.37 ms; 2%; n = 21; P = 0.23; Wilcoxon-matched pairs test). In contrast, offset latency increased from 38.81±1.57 to 46.96±1.02 ms (21%; n = 21; P<0.001; Wilcoxon-matched pairs test).

Whisker response magnitude and duration increased in infra- and supragranular layers (Fig 10B). In infragranular layers, the first component did not increase significantly (from 0.78±0.2 to 0.83±0.2 spikes/stimulus; 6%; n = 23; P = 0.07; Wilcoxon-matched pairs test). Spikes in the second component of the response were increased from 1.06±0.3 to 1.22±0.3 spikes/stimulus (15%; n = 23; P<0.001; Wilcoxon-matched pairs test; Fig 10B). In supragranular layers, the first component was not affected (from 0.73±0.1 to 0.79±0.1 spikes/stimulus; 8%; n = 22; P = 0.062; Wilcoxon-matched pairs test) while the second component was increased (0.68±0.1 to 0.81±0.1 spikes/stimulus; 19%; n = 22; P<0.001; Wilcoxon-matched pairs test; Fig 10B).

In accordance with previous studies [40], we confirm that L1 exerts an inhibitory influence on whisker responses. Our results demonstrate that GABAergic inhibitory transmission in L1 is implicated in the regulation of cortical excitability and sensory response magnitude and duration.

### L1 GABAergic system is crucial in sensory cortical regulation by POm

**Fig 11:**
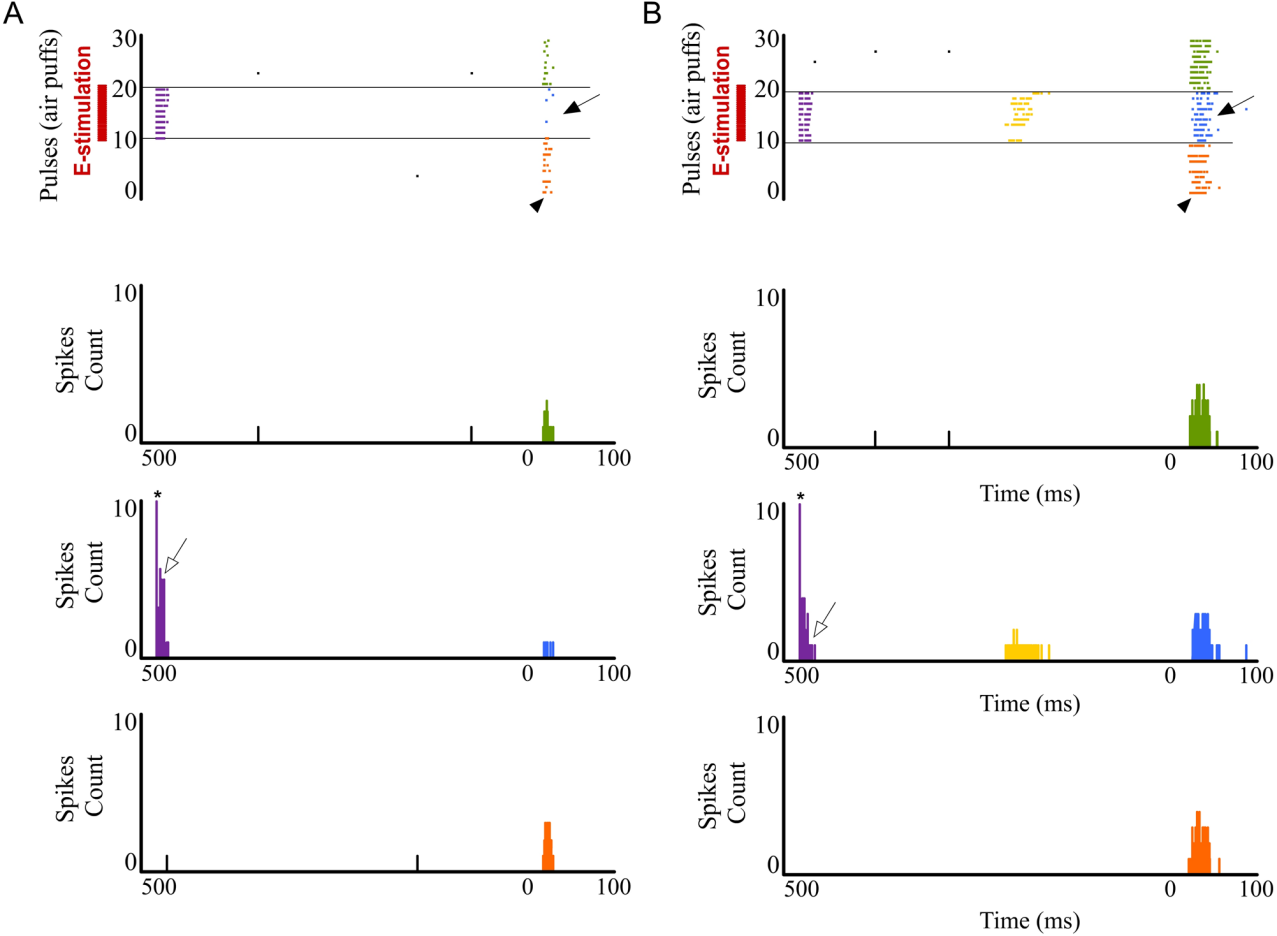
POm E-stimulation before whisker stimulus does not decrease cortical response magnitude and duration when PTX is applied in L1. Raster plots and PSTHs are shown for a supragranular neuron before (A) and after (B) GABAergic inhibitory inactivation in L1. Before PTX application, sensory response (filled arrows) of this example neuron was abolished by POm E-stimulation before whisker stimulus. However, POm E-stimulation did not reduce sensory response when GABAergic inhibitory transmission in L1 was inactivated (B). GABAergic inactivation in L1 allowed POm E-stimulation to cause rebound spikes (in yellow). PTX effect is also shown in the sensory response (arrowheads) enlargement after PTX application. Control condition before (orange) and after (green) POm E-stimulation condition (blue) are shown. POm E-stimulation was applied 500 ms before pulses (air puffs) 11 to 20. Open arrows indicate orthodromic spikes elicited by POm E-stimulation. * indicates stimulation artifacts.

**Fig 12:**
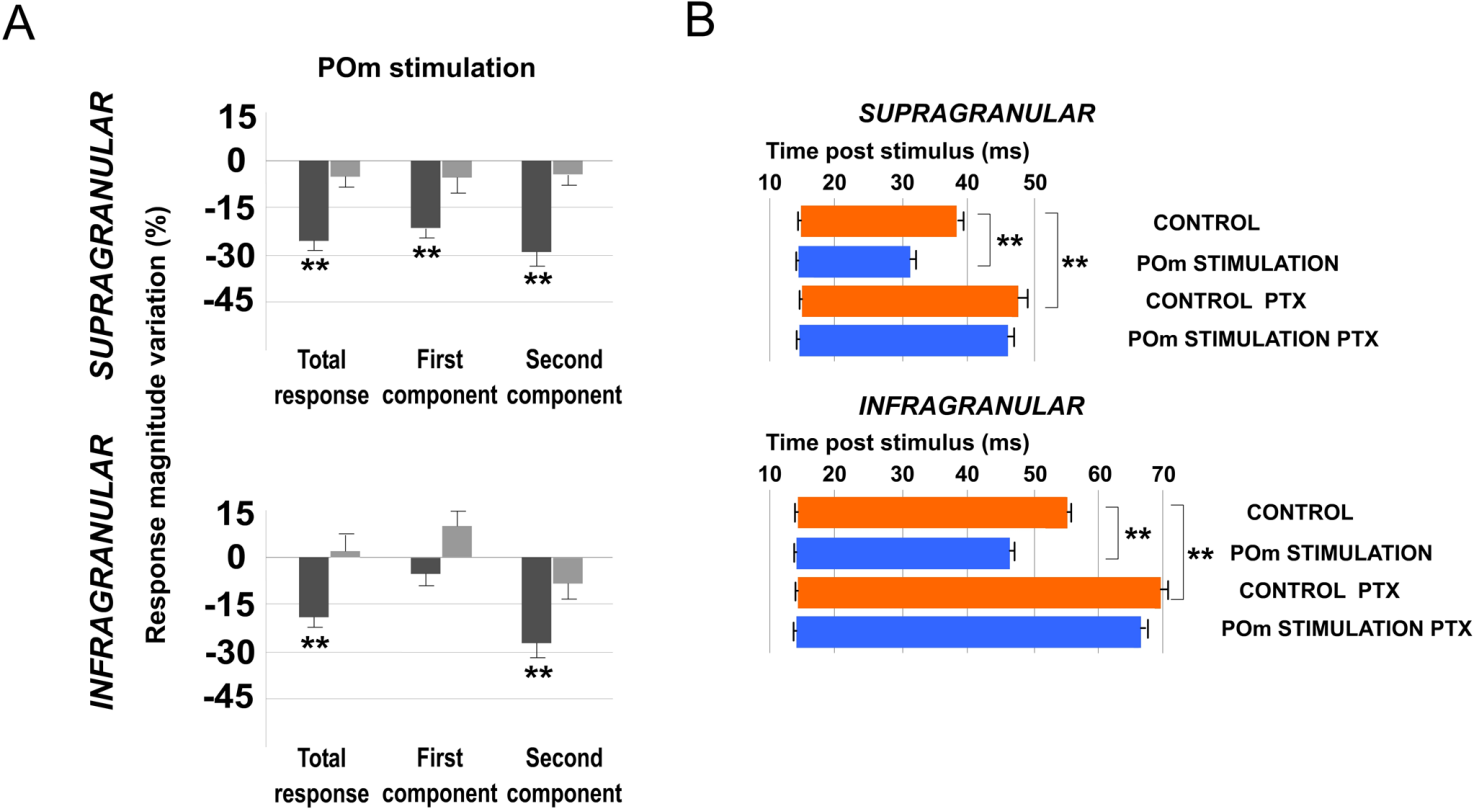
POm E-stimulation before whisker stimulus does not decrease cortical response magnitude and duration when PTX is applied in L1. (A) Percentage change in mean response magnitude by POm E-stimulation before (black) and after PTX application (grey). POm E-stimulation did not decrease cortical response magnitude when GABAergic inhibitory transmission in L1 was blocked. (B) PTX application in L1 increased whisker offset response latency in infra- and supragranular layers. POm E-stimulation before whisker stimulus did not decrease cortical response duration when PTX was applied in L1. Control (orange) and POm E-stimulation (blue) conditions are shown.

Next, in that condition of L1 inhibitory transmission inactivation by PTX, we applied E-stimulation to the POm before (500 ms) each whisker stimulus (Fig 11). We found that response magnitude did not significantly decrease by POm E-stimulation (infragranular layers: 1%; n = 22; P = 0.67; Wilcoxon-matched pairs test; in supragranular layers: -4%; n = 21; P = 0.18; Wilcoxon-matched pairs test; Fig 12A, Total response). Response duration was also not affected by POm E-stimulation in this condition. Offset latencies were not reduced (in infragranular layer: -5%; n = 22; P = 0.098; Wilcoxon-matched pairs test; and in supragranular layer: -3%; n = 21; P = 0.9; Wilcoxon-matched pairs test; Fig 12B). Onset latencies in both layers were not significantly affected.

#### Blocking P/Q-type Ca2+ channels in L1 prevents POm electrical stimulation effect

**Fig 13:**
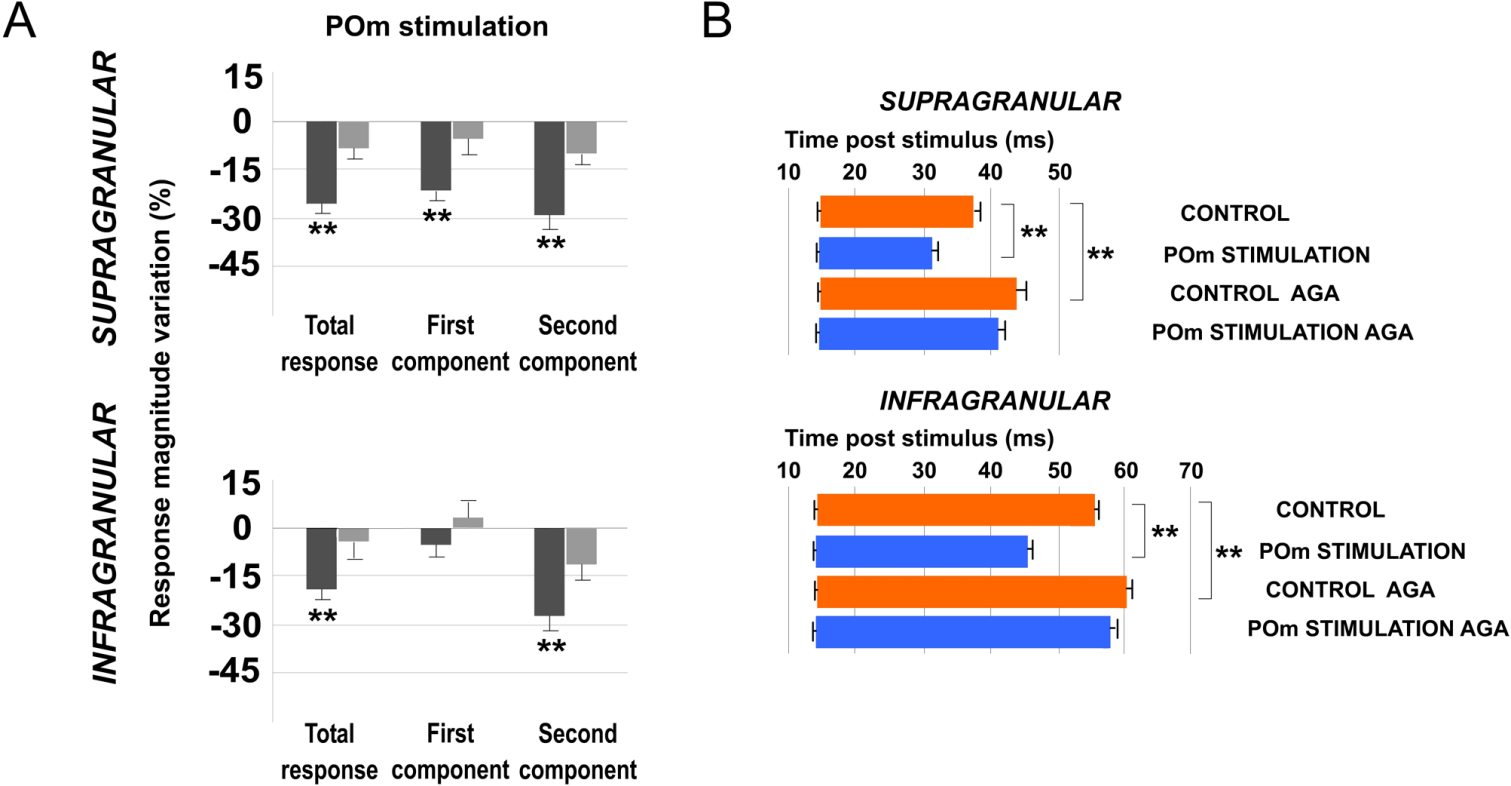
POm E-stimulation before whisker stimulus does not decrease cortical response magnitude and duration when AGA is applied in L1. (A) Percentage change in mean response magnitude by POm E-stimulation before (black) and after AGA application (grey). POm E-stimulation before each stimulus (500 ms) did not decrease cortical responses when P/Q-type voltage-gated calcium channels were blocked. (B) AGA application in L1 increased whisker offset response latency in infra- and supragranular layers. POm E-stimulation did not decrease cortical response duration when P/Q-type voltage-gated calcium channels were blocked. Control (orange) and POm E-stimulation (blue) conditions are shown.

When we applied POm E-stimulation before (500 ms) whisker stimulus in P/Q-type voltage-gated calcium channels blocked condition we found that cortical sensory responses did not significantly decrease. Response magnitude did not significantly decrease by POm E-stimulation in infragranular layers (-3%; n = 23; P = 0.15) and in supragranular layers (-6%; n = 22; P = 0.09; Wilcoxon-matched pairs test; Fig 13A Total response). Response duration was also not affected by POm E-stimulation in this condition. In infragranular layers, offset latencies were not reduced (-3%; n = 23; P = 0.39; Wilcoxon-matched pairs test) and the same was found in supragranular layers (-6%; n = 22; P = 0.08; Wilcoxon-matched pairs test; Fig 13B). Onset latencies in both layers were not affected.

#### L1 E-stimulation just before whisker stimulus modulates sensory response magnitude and duration in barrel cortex

**Fig 14:**
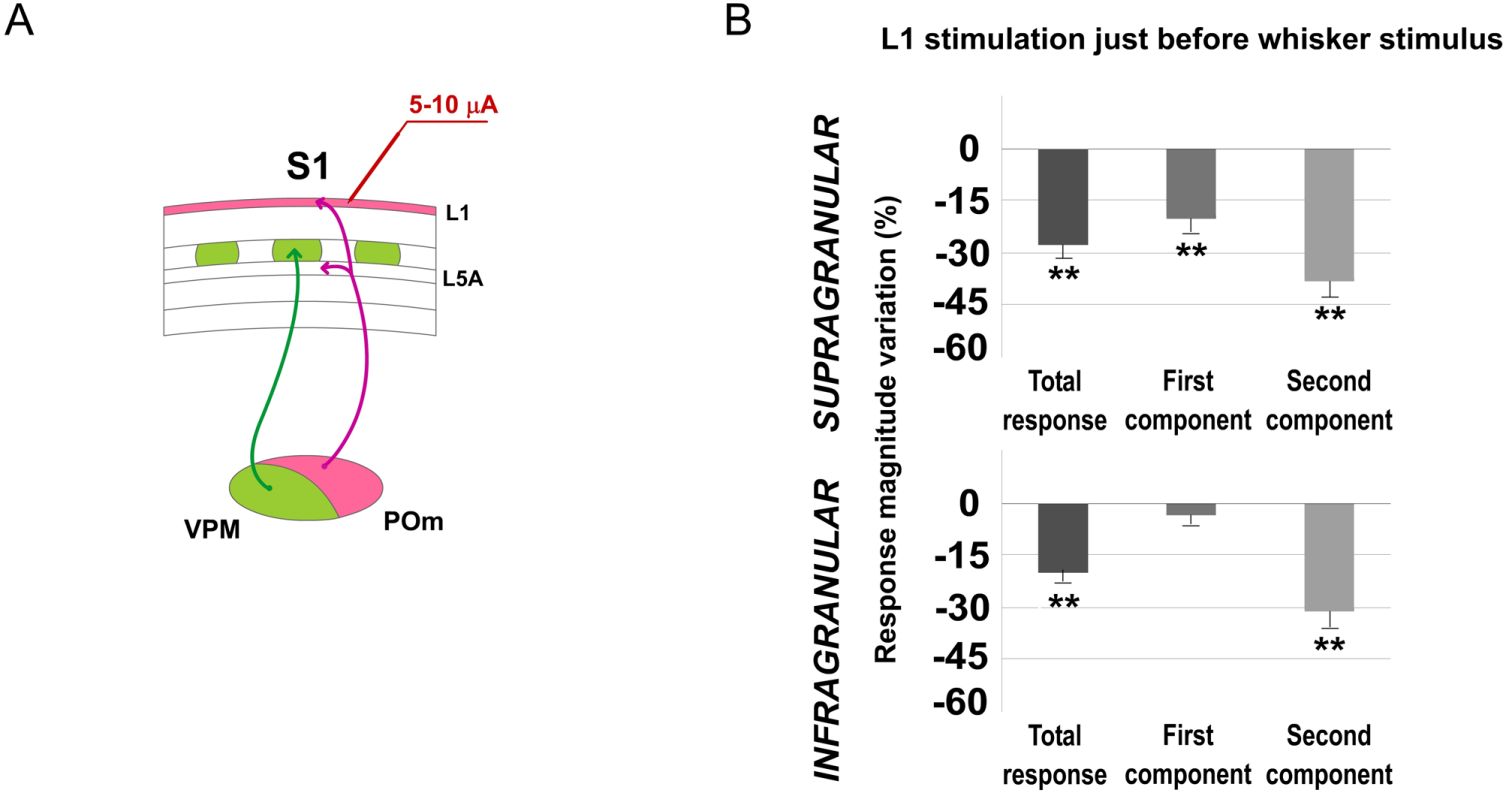
L1 E-stimulation before sensory stimulus modulates cortical responses. (A) Schematic diagram indicating the experimental manipulation of the barrel cortex L1. (B) Mean response magnitude variation (%) by L1 E-stimulation is quantified in this Fig Magnitude reduction was more prevalent in the second component of the response in both layers. In infragranular layers, in the first component we did not find a significant decrease of spikes. However, spikes in the second component were decreased strongly by L1 E-stimulation. In supragranular layers, in both components we found a significant reduction of spikes.

To further confirm whether the observed POm modulation of cortical responses was mediated by L1, we investigated cortical response changes by applying L1 E-stimulation in S1 before sensory stimulus in 6 rats. Similar to POm E-stimulation, L1 E-stimulation (single pulse of 5–10 μA, 0.5 ms) before (150 ms) each whisker stimulus was accompanied by a marked change in cortical sensory responses. Magnitude and duration of cortical responses significantly decreased. Again, magnitude reduction was more prevalent in the second component of the response (Fig 14). In infragranular layers, the first component was not affected (from 1.11±0.1 to 1.08±0.1 spikes/stimulus; -3%; n = 33; P = 0.67). Evoked spikes were decreased in the second component of the response by L1 E-stimulation from 0.96±0.2 to 0.67±0.1 spikes/stimulus (-30%; n = 33; P< 0.001). In infragranular layers, 72% of neurons (33 of 46) displayed significant changes in responses correlated with L1 E-stimulation. In supragranular layers both response components were affected. The first component decreased from 1.02±0.1 to 0.83±0.1 spikes/stimulus (-19%; n = 39; P< 0.001) and from 0.78±0.1 to 0.48±0.1 spikes/stimulus (-38%; n = 39; P<0.001) in the second component. A total of 85% of supragranular layer neurons (39 of 46) displayed changes correlated with L1 E-stimulation.

The latency of the response onset did not change in infragranular neurons (13.82±0.13 ms in control and 13.68±0.1 ms after L1 E-stimulation; -1%; n = 33; P = 0.62). However, as occurred in POm E-stimulation condition, the main effect was found in offset latency (from 55.18±1.04 to 51.21±1.1 ms; -7%; n = 33; P<0.001), decreasing the duration of the response. The latency of the response onset was reduced in supragranular neurons from 14.67±0.3 to 13.93±0.25 ms (-5%; n = 39; P = 0.002) and offset latencies decreased from 40.58±1.54 to 34.33±1.4 ms (-15%; n = 39; P<0.001), as well.

These results were similar to POm E-stimulation suggesting that the observed effects produced by POm E-stimulation in sensory cortical responses were mainly mediated by L1.

### POm controls the sensory processing in S2 and this regulation is modulated by corticofugal activity from L5 in S1

POm neuron projections also target other cortical areas including S2 [18, 21]. It is well described the reciprocal connections between these areas. The above results demonstrate that POm modulates magnitude and duration of S1 cortical responses to sensory input. This sensory response adjustment could be also present in the processing of information between somatosensory cortical areas. Then, to determine the possible role exerted by the POm in the adjustment of somatosensory cortical processing between S1 and S2, we performed a complementary set of experiments investigating whisker response changes in S2 by electrically stimulating S1 and by muscimol-induced inactivation of the POm. The following results describe below demonstrate that POm activity is also controlling the sensory processing in S2 and this regulation is modulated by corticofugal activity from L5 in S1.

#### L5 E-stimulation in S1 before sensory stimulus modulates whisker response in S2

It is known that L5 corticofugal neurons in S1 project to the POm (see Introduction). From here, POm neuron projections also target other cortical areas including higher-order somatosensory cortical regions.

**Fig 15:**
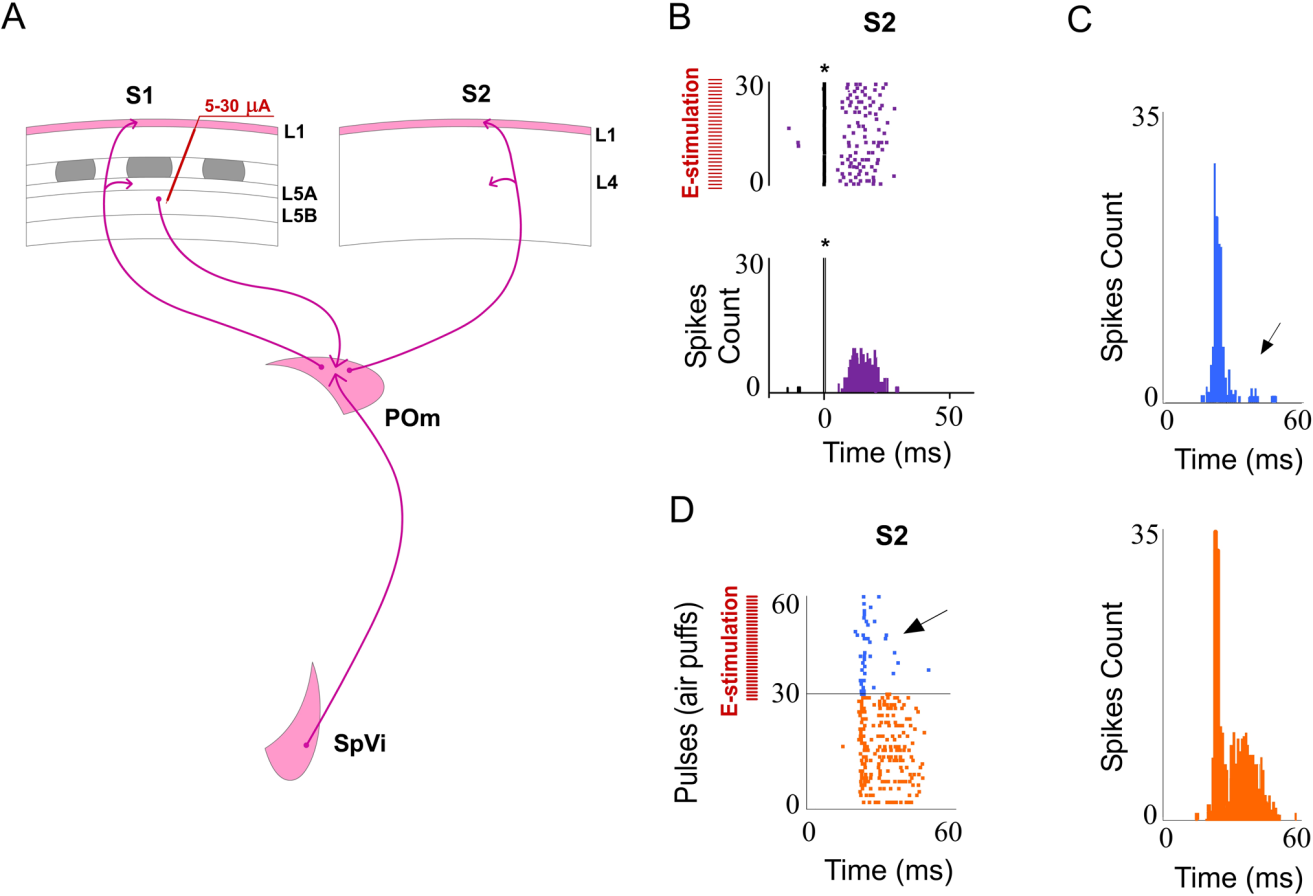
L5 E-stimulation in S1 before sensory stimulus modulates S2 whisker responses. (A) Schematic diagram summarizing the corticothalamocortical circuitry from S1 to S2 through the POm. The experimental manipulation of the barrel cortex is also shown. (B) An example of S2 evoked orthodromic spikes by L5 E-stimulation in barrel cortex is shown. * indicates stimulation artifacts. (C, D) L5 E-stimulation in barrel cortex just before whisker stimulus reduced responses in S2. Raster plots (D) and PSTHs (C) are shown for a sample infragranular response. L5 E-stimulation in S1 shortened responses and reduced spikes mainly in the second response component (arrows). Control (orange) and POm E-stimulation (blue) conditions are shown. Spikes are aligned on sensory stimulus (air puff) presentation (Time 0 ms). L5 E-stimulation in S1 was applied 150 ms before air puffs (31 to 60 pulses; red bars).

**Fig 16:**
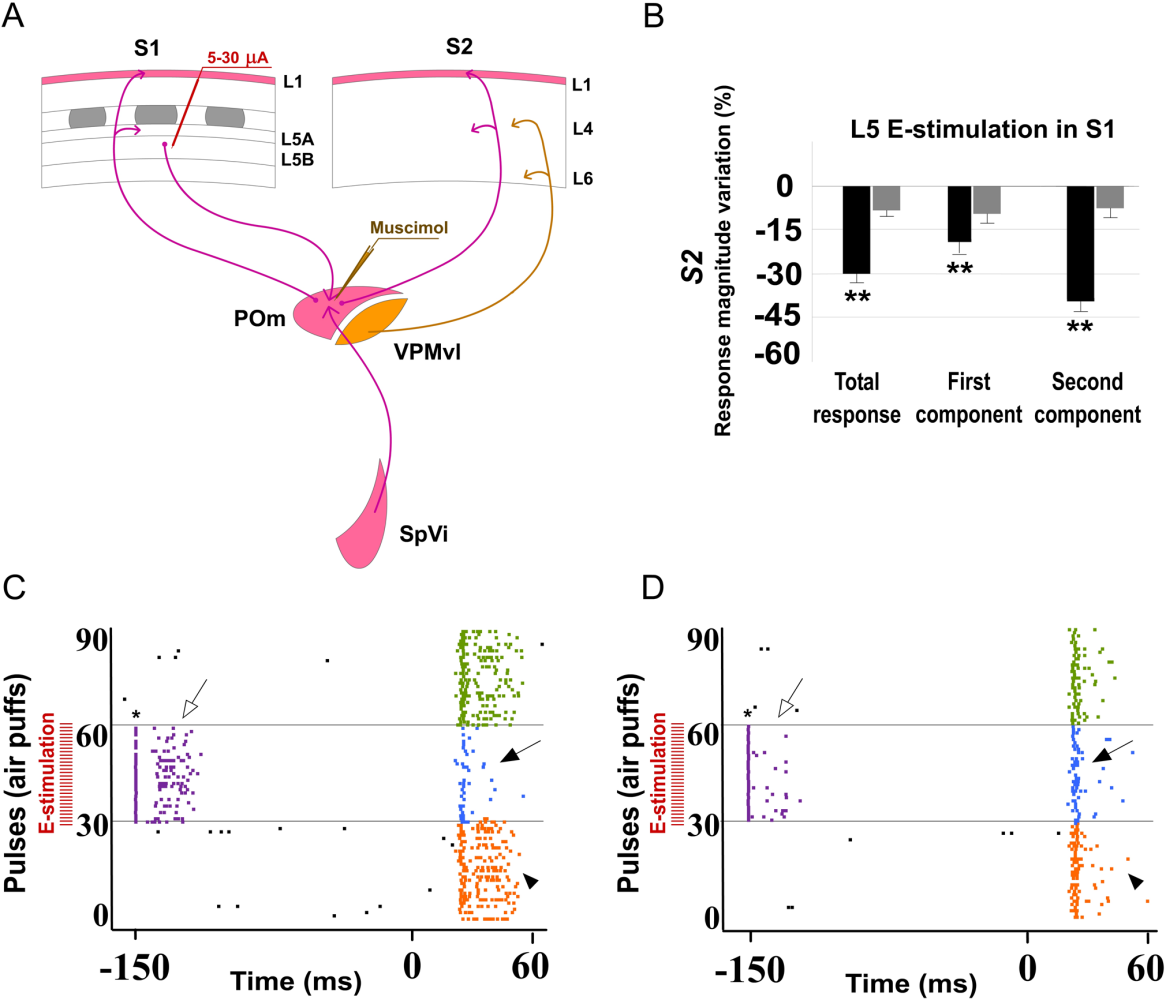
L5 E-stimulation in S1 does not modulate sensory responses in S2 when POm is inactivated. (A) Schematic diagram summarizing the corticothalamocortical circuitry from S1 to S2 through the POm and the extralemniscal pathway to S2 from the VPMvl thalamic nucleus. The experimental manipulation of the barrel cortex and POm is also shown. (B) Change (%) in mean S2 sensory response magnitude by stimulating L5 of S1 before (black) and after POm muscimol inactivation (gray). L5 E-stimulation in S1 did not reduce significantly sensory response spikes in S2 when POm was inactivated. ** P<0.001. (C) Sensory responses in S2 were reduced when we applied E-stimulation in L5 of S1 before each stimulus (filled arrows). This effect was abolished when the POm was inactivated with muscimol (D). Control condition before (orange) and after (green) POm E-stimulation condition (blue) are shown. E-stimulation was applied 150 ms before pulses 31 to 60. Spikes evoked in S2 by E-stimulation of L5 in S1 were eliminated by POm inactivation (open arrows). At the stimulation intensities used in this experiment, we have not observed antidromic activation in S2. Sensory responses were significantly decreased after muscimol application but not totally eliminated. Only spikes in the second component of the response were abolished (arrowheads). Spikes in the first component were not reduced by POm inactivation. * indicates stimulation artifacts.

We recorded vibrissal responses in the whisker representation area of S2 in 11 rats. We found that S2 neurons displayed a low firing rate in spontaneous conditions (0.87±0.6 spikes/s; n = 40) and displayed a contralateral response to whisker displacements. Then, we investigated sensory response changes in S2 neurons by L5 E-stimulation in S1 before whisker stimulus (150 ms). S1 L5 E-stimulation alone (single pulse of 0.5 ms; 5–30 μA) elicited strong activity in S2 (Fig 15B). The latencies of these evoked spikes varied in the range of 8–40 ms (mean latency: 21.61±0.7 ms; n = 40). When we stimulated electrically the L5 of S1 before each sensory stimulus, response magnitude decreased from 1.95± 0.3 to 1.35± 0.2 spikes/stimulus (-30%; n = 36; P<0.001). A total of 90% of S2 recorded units (36 of 40) displayed reduction in responses correlated with L5 E-stimulation in S1. First and second component results are described and quantified in Fig 16B. The latency of the response onset did not change (from 14.45±0.1 to 14.25±0.13 ms; -1%; n = 36; P = 0.31) but offset latency decreased by L5 E-stimulation (from 44.33±0.21 to 35.63±0.46 ms; -20%; n = 36; P<0.001).

#### Cortico-cortical sensory processing adjustment is abolished when POm is inactivated with muscimol

Next, to demonstrate that POm was implicated in the effects described above, we inactivated the POm by infusing a small volume (0.1–0.3 μl; 1 mM) of muscimol. We found that 15 min after muscimol application, L5 E-stimulation in S1 could not reduce sensory response spikes in S2 (Fig 16). Change in mean sensory response magnitude by stimulating L5 of S1 before and after POm muscimol inactivation is quantified in Fig 16B. These findings indicate that POm activity is also controlling the sensory processing in S2 and this regulation is modulated by corticofugal activity from L5 in S1.

Furthermore, we found that S2 robust activity in response to L5 E-stimulation in S1 alone was eliminated after POm inactivation (Fig 16C and 16D white arrows) with a subsequent return after washout (data not shown). This finding is in agreement with other studies on corticothalamocortical communication implicating the POm in information transfer to higher-order cortical areas [25, 41, 42].

In sum, our results demonstrate that POm is implicated in the adjustment of information processing between somatosensory cortical areas.

## Discussion

Here, using a combination of electrophysiology and pharmacology *in vivo*, we show that POm modulates magnitude and duration of supra- and infragranular barrel cortex whisker responses. Our findings demonstrate that L1 inputs from POm impose a time and intensity dependent regulation on cortical sensory processing. Moreover, we found that L1 GABAergic system mediates this process and that blocking P/Q-type Ca2+ channels in L1 prevents POm adjustment of whisker responses in the barrel cortex. Additionally, we found that POm is also controlling the sensory processing in S2 and this regulation is modulated by corticofugal activity from L5 in S1. Taken together, our data demonstrate the determinant role exerted by the POm in the adjustment of somatosensory cortical processing and in the regulation of cortical processing between S1 and S2. We propose that this adjustment could be a thalamocortical gain regulation mechanism also present in the processing of information between cortical areas.

### Antidromic or rebound activities are not implicated in our thalamic E-stimulation effects

It is known that low intensity thalamic E-stimulation strongly activates thalamocortical neurons [45]. Yang and collaborators demonstrated that thalamic E-stimulation was capable of eliciting a cortical response that resembles the cortical activity pattern evoked by a whisker stimulus [59]. Their E-stimulation protocol (single current pulse; 10–150 μA, 100 μs duration) activated only a small region of thalamic tissue. Intensity used in our experiments (<80 μA) was estimated to activate neurons within a maximal radius that would not exceed 0.5 mm [46], suggesting that the effect induced by the E-stimulation was likely concentrated around the stimulation site. In our experiments, no cortical evoked responses were elicited when the thalamic E-stimulation was performed outside the POm or VPM. In these cases, we did not observe any detectable changes in cortical sensory responses by thalamic E-stimulation (data not shown). We assume that thalamic E-stimulation minimally affects neighbouring structures, however because POm and VPM are immediately adjacent to each other, we can not rule out possible mixed effects between VPM and POm E-stimulation. To clarify this issue we performed a set of complementary studies. Muscimol inactivation of these nuclei in separate experiments demonstrated different thalamic influence in cortical processing. VPM inactivation by muscimol abolished whisker responses. However, POm inactivation enhanced spontaneous activity and whisker responses. Moreover, it is known that L1 receives synaptic inputs from POm but not from VPM or L4. We found that E-stimulation of L1 or POm caused similar effects in cortical sensory responses. These findings together with electrode tip position on histological sections allow us to discriminate E-stimulation effects and to understand the different function of these nuclei on cortical processing. Moreover, it is known that VPM lesions abolished the cortical responses evoked by whisker stimulation [59]. Therefore, in our POm inactivation experiments a further indication that the muscimol did not affect the VPM was the increase of cortical whisker responses.

We did not find rebound activity induced by POm or VPM E-stimulation within the current range used (<80 μA). However, we found excitatory rebound activity in some cases applying VPM E-stimulation with a higher intensity (>150 μA) (data not shown). POm E-stimulation did not elicit excitatory rebound activity even at 150 μA (data not shown). We only found excitatory rebound activity at the intensities used in our studies (<80 μA) when GABAergic inhibitory transmission in L1 was blocked by PTX. GABAergic inactivation in L1 increased whisker response magnitude, increased basal activity and allowed POm E-stimulation to cause rebound spikes in some cases as shown in Fig 11B. Moreover, in our experiments we used different time intervals between POm E-stimulation and sensory stimulus ranged from 50 to 1000 ms. We consider that the time of these intervals is both, variant and long enough to allow rebound activity to be detected. However, we did not find it, ruling out rebound activity implication in our thalamic E-stimulation effects. We consider that our results support an absence of implication of POm adaptation in our E-stimulation results. For example, sensory cortical whisker responses were strongly reduced at all intervals (1–20 Hz) in supragranular layers (Fig 6). Yet infragranular responses were not significantly reduced by POm E-stimulation at 20 Hz, a frequency high enough to cause adaptation. Furthermore, L1 E-stimulation induced similar cortical effects. In agreement with that, POm E-stimulation before whisker stimulus did not reduce cortical sensory response when GABAergic inhibitory transmission in L1 was inactivated.

Anatomically, POm receives corticothalamic inputs from L5, thus, infragranular layers activity elicited by POm E-stimulation could also result from antidromic activation of corticothalamic neurons and their axon collaterals. Thus, E-stimulation of the thalamus that is intended to activate thalamocortical afferents may also produce antidromic activation of corticothalamic neurons that subsequently contributes, via axon collaterals, to the synaptic response in the infragranular layers. Cortical studies have demonstrated that orthodromic stimulation effects are stronger than antidromic effects even between areas with strong direct projections [68–70]. Previous thalamocortical studies demonstrated that the threshold for antidromic activation was significantly higher than for orthodromic activation [45, 71]. Rose and Metherate found that mean orthodromic cortical response threshold from stimulating thalamic afferents was 28 μA. Antidromic stimulation of corticothalamic projections resulted in a mean threshold of 214 μA. This implies that low-current thalamic stimulation activates relatively few corticothalamic neurons and that it can strongly activate thalamocortical neurons. Furthermore, the threshold for evoking an antidromic spike in pyramidal neurons by L1 E-stimulation is higher than the threshold required to elicit synaptic responses in the same neuron [40]. In our experiments, we did not observe antidromic activation in infra- or supragranular recordings at stimulation intensities used in these experiments. Antidromic contribution to our findings was therefore ruled out.

### L1 implication in POm control of cortical sensory responses

L1 is an important site of integration as it contains feedback corticocortical inputs from other cortical areas and TC inputs mainly from high order nuclei. In our experiments, we found that L1 inputs from POm impose precise regulation on cortical processing. In some of our experiments, we used PTX to block GABAergic transmission in L1, as was also used in other recent cortical studies *in vitro* [74] and *in vivo* [75, 76]. We also use AGA to block P/Q-type Ca2+ channels [63–65]; see below). Our results showed that POm E-stimulation before whisker stimulus did not reduce cortical sensory responses when PTX or AGA was applied over cortical surface. We did not try to determine whether these drugs reached other cortical layers, which could have directly inactivated inhibitory influence in those layers. However, we found in our experiments that POm E-stimulation did not reduce sensory responses in infra- and supragranular layers within a few minutes (<5 min) of the PTX or AGA application over the cortical surface. Taking into account that this effect was produced rapidly at the same time in both layers and since the diffusion of the drug into the infragranular layers requires more time, we consider these effects to be induced mainly by L1.

These results were similar to those resulting from POm inactivation. Furthermore, similar to POm E-stimulation, L1 E-stimulation before sensory stimulus also reduced responses in infra- and supragranular layers. It is known that L1 E-stimulation evokes two types of laminar activity in barrel cortex depending on intensity [40]. At lower intensities (<10 μA) the synaptic activation evoked by this E-stimulation was restricted to L1 and upper L2. In contrast, at higher intensities (>10 μA) L1 E-stimulation activated the entire cortical column. In our L1 E-stimulation protocol, we applied low intensities (<10 μA) to examine the effect of L1 activation on whisker responses. We can not rule out the possibility that in our experiments L1 E-stimulation activated other cortical layers. Even L1 E-stimulation can antidromically activate vertically projecting axons of Martinotti interneurons inducing effects in other layers [40, 77]. Since, we found in our experiments similar cortical effects induced by POm E-stimulation and by L1 E-stimulation, we consider ruling out these possibilities.

In the rat barrel cortex, the border between L5 and L6 has been described at depths of 1400–1600 μm [24, 78]. In our experiments, neurons were recorded in depths from 200 to 600 μm and from 900 to 1500 μm. According to this anatomical data, we must consider that infragranular neurons recorded in our experiments were mainly from L5. Since POm strongly innervates L5A [18, 21], we considered to separate our infragranular recordings in two groups according to the depths of the recordings. A preliminary analysis of single-units from both groups (superficial and deep recordings) showed similar quantitative modulation by POm manipulations. L5A and L5B pyramidal neurons have an apical dendrite reaching L1. In accordance with that, our findings show that POm may exert its control of cortical sensory responses mainly through L1. This layer also contains a dense plexus of apical dendrites of supragranular pyramidal neurons but not of granular neurons [60, 79]. One remaining unknown is the function of L5A inputs from POm.

### POm modulates the temporal integration window of cortical sensory responses

Recent studies suggest that POm projections make excitatory synapses with barrel cortex pyramidal cells [20, 39, 43, 73]. According to them, in our experiments, POm E-stimulation alone elicited orthodromic spikes in infra- and supragranular layers of barrel cortex. However, our results also showed that POm E-stimulation just before sensory stimulus reduced magnitude and duration of cortical whisker responses. Moreover, unexpectedly, we found that POm inactivation by muscimol caused an enhancement of both sensory cortical responses and spontaneous cortical activity in the barrel cortex suggesting that POm is tonically regulating cortical excitability in this region. How can these intriguing effects be explained? Our findings show that POm exerts its control of cortical sensory response magnitude and duration using the GABAergic inhibitory system in L1. Therefore, L1 inhibitory interneurons are other potential targets of POm projections. In the mouse prefrontal cortex, a recent study described that matrix thalamocortical projections terminate in outer L1, and their activation drives robust synaptic responses in L1 interneurons [80]. They found that L1 thalamocortical projections preferentially drove inhibitory interneurons of L1 and were much more effective at exciting L1 interneurons than L2/3 pyramidal cells. Accordingly, it is known that L1 inputs generate direct, rapid excitatory postsynaptic potentials in L1 interneurons [60, 85]. These interneurons could rapidly truncate afferent excitation of infra- and supragranular pyramidal neurons, limiting the temporal window during which action potentials can be generated. Our results are also in agreement with that idea. We found that POm E-stimulation or L1 E-stimulation reduced spikes mainly in the second response component. Therefore, this interplay between excitation and inhibition at the level of the barrel cortex could provide a “window of opportunity” for generating cortical responses. Our findings are consistent with that hypothesis. As our results show, the duration of the responses is regulated by POm activity. L1 inputs from POm could activate L1 GABAergic interneurons strengthening cortical inhibition, which shortens the response window. We found that increasing POm E-stimulation intensity reduced more strongly the duration of cortical responses (see Results; Fig 8). A relevant assumption supported by our data is that prolonged response duration (prolonged window) was observed when GABAergic inhibitory transmission in L1 was blocked (Fig 12B).

Accordingly, we found that response magnitude and duration of cortical neurons changed by POm E-stimulation intervals before sensory stimulus (described in Fig 6). Therefore, that interval determines the outcome of the interaction. Recently, both anatomical and physiological findings have shown that ascending inputs from the brainstem and descending inputs from L5 converge on single thalamocortical neurons in POm [25]. Both individual pathways interact functionally in a time-dependent manner and when co-activated, increase the output of thalamus supralinearly [25]. Moreover, Shlosberg et al. found that when pairing L1 E-stimulation with whisker deflection, the interval between the stimuli determined the outcome of the interaction, with facilitation of sensory responses dominating the short (<10 ms) intervals and suppression prevailing at longer (>10 ms) intervals [40]. Then, same effects could be induced by POm E-stimulation using those intervals.

We propose this mechanism could allow the temporal cortical integration of inputs from distinct pathways and could act to “reset” the network to generate the next cortical response avoiding the somatosensory cortex be captured by a single stimulus.

Since it is well described that POm is involved in temporal processing related to sensory-motor control of whisker movement [17, 34, 35], it is then possible that this mechanism could play a crucial role in sensory-motor interaction allowing the POm to control the temporal integration of the incoming tactile information during whisking exploration. The accuracy of whisking could be controlled by POm activity to optimize sensory processing. Accordingly, it has been suggested that the whisker sensory-motor system is involved in closed-loop computations [94, 95]. In particular, single unit responses from whisker sensory and motor areas show generic signatures of phase-sensitive detection and control at the level of thalamocortical and corticocortical loops [94, 95]. These loops are likely to be components within a greater closed-loop vibrissa sensory-motor system, which optimizes sensory processing. Our results are in agreement with that proposal.

### Possible implication of parvalbumin interneurons in POm control of cortical responses

L1 inhibitory interneurons provide a direct source of apical dendritic inhibition to supra- and infragranular layer pyramidal neurons [80–82]; and also form inhibitory synapses onto other L1 interneurons and L2/3 interneurons [83–86]. Interneurons of L1 are heterogeneous [60, 79, 87–90]. To study in more detail the L1 inhibitory implication in POm control of cortical processing, we applied Cav2.1 (P/Q- type) voltage-gated calcium channels blocker and found that blocking P/Q-type Ca2+ channels avoided POm E-stimulation effects. It is known that these channels are expressed on parvalbumin (PV) interneuron axon terminals and mediate GABA release from fast spiking interneurons to pyramidal cells [63–65]. Consequently, it is possible that presumed PV+ interneurons were implicated in a dynamic control of sensory cortical processing by POm. Other studies have demonstrated that PV+ interneurons participate in control gain of sensory responses [86, 91, 92]. Furthermore, recent findings demonstrate that the conditional ablation of Cav 2.1 channel function from cortical PV+ interneurons alters GABA release from these cells, impairs their ability to constrain cortical pyramidal cell excitability [93].

### The main effect of POm manipulation occurs in the second component of cortical response: possible NMDA receptors implication and cortical plasticity

It is known that short-latency spikes evoked by whisker stimulation in the barrel cortex are mainly mediated through non-NMDA receptors while NMDA receptors are implicated mainly in spikes generated later after them [72]. Studies from our laboratory confirmed the implication of NMDA receptors in the late component of cortical tactile responses [57, 58]. A recent study suggest that POm associated synaptic pathways in barrel cortex are responsible for these mediating whisker-evoked NMDA receptor dependent spikes [73], in agreement with our results. Since these receptors have been directly implicated in cortical synaptic plasticity, our findings have important consequences in sensory processing implicating the POm in the control of cortical synaptic plasticity by reducing the time-window of activation in cortical neurons.

### POm implication in the regulation of cortical processing between S1 and S2

**Fig 17:**
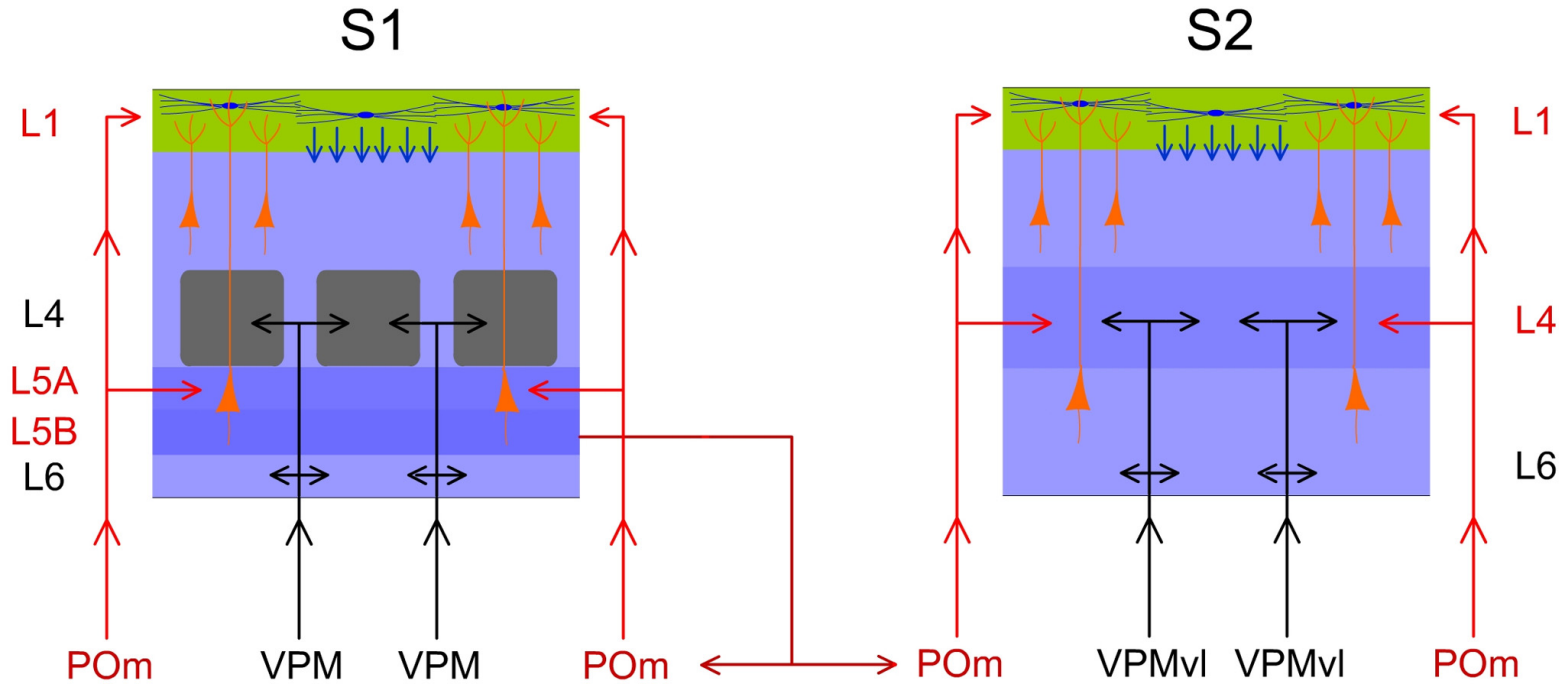
POm influence on somatosensory cortical response modulation. Schematic diagram summarizing the ascending thalamocortical pathways to S1 and S2. Corticothalamocortical circuitry from S1 to S2 through the POm is also shown. L5B corticofugal neurons in S1 project to the POm [9, 15, 25, 26, 43]. Ascending inputs from the brainstem and descending inputs from L5 converge on single thalamocortical neurons in POm [25]. Both individual pathways interact functionally in a time-dependent manner [25]. From here, POm neuron projections also target S2 [18, 21]. POm is also controlling the sensory processing in S2 and this regulation is modulated by corticofugal activity from L5 in S1 [25]. Whisker-evoked sensory information is rapidly relayed to L1 neurons, which, in turn, act to powerfully inhibit whisker-evoked responses [40, 60]. In accordance with previous studies [40], we confirm that L1 exerts an inhibitory influence on whisker responses.

Our results demonstrate the determinant role exerted by the POm in the adjustment of somatosensory cortical processing in S1 and S2. We found that vibrissal stimulus responses recorded in S1 and S2 were modulated in magnitude and duration by POm activity. These effects were abolished when we inactivated the POm with muscimol. Since our results show that POm exerts its control of barrel cortex sensory responses mainly using L1 GABAergic system, it is then possible that the same mechanism could be used by the POm to regulate sensory responses in S2 (Fig 17). Accordingly, strong POm connections to L1 in S2 have been described [18, 96].

However, in contrast to S1, it is known that L4 of S2 receives a strong projection from the POm [39, 43]. In agreement with that, in our experiment, whisker sensory responses in S2 were reduced after POm inactivation (for example see Fig 16D, arrowheads). Spikes in the second component of the response were abolished. However, spikes in the first component were not reduced by POm inactivation suggesting they come from a different pathway. Ascending whisker signals reach S2 not only through the POm. It is known that S2 receives (focally in L4 and L6; extralemniscal pathway) from thalamocortical neurons located in the ventrolateral part of the VPM [66, 67]. Since this pathway should not be affected by POm inactivation in our experiments, it is then possible that spikes in the first component of S2 whisker responses were caused by extralemniscal inputs. The short latencies of these spikes rule out the possible VPM-S1-S2 route.

We show in our experiments that vibrissal stimulus responses recorded in S2 were reduced in magnitude and duration when we applied E-stimulation in L5 of S1 before the whisker stimulus. It is possible that L6 neurons, which send feedback inputs to thalamus, were also affected by L5 E-stimulation. However, a recent study demonstrates that stimulation of L6 does not activate S2 via this circuit [43]. In our experiments, this cortical sensory processing adjustment between S1 and S2 was abolished when POm was inactivated with muscimol. L5 E-stimulation in S1 could not reduce sensory response spikes in S2 after POm inactivation (Fig 16). Furthermore, we found that S2 robust activity in response to L5 E-stimulation in S1 alone was eliminated after POm inactivation. This finding is in agreement with other studies on corticothalamocortical communication implicating the POm in information transfer to higher-order cortical areas [25, 41, 42].

### POm activity modulation of cortical processing. Functional implication

There is a huge range of stimuli that reach the cortex, each with different intensities and durations. To process them the system must have the capacity to regulate itself to detect the weakest ones and not be saturated by the strongest ones. This allows the system to process a wider range of stimuli improving the ability to detect and identify tactile features. Based on our findings, we propose that control of cortical sensory processing exerted by POm could be part of a mechanism that has the ability to regulate the processing gain, depending on the relative intensities of stimuli across the entirety of vibrissae space. This integration of multi-whisker activity could be achieved by the POm and transmitted to the cortex to adjust the sensory processing.

Sensory activity carried by this pathway could allow the adjustment of the specific sensory content processing in the cortex. Our results show that there is a fundamental difference between the lemniscal and paralemniscal thalamic nuclei in terms of cortical influence. We must consider that these parallel pathways have a complementary function in sensory processing. Lemniscal and paralemniscal parallel ascending projection systems from the thalamus could convey specific sensory content and stimuli global sensory activity, respectively. Global activity carried by the paralemniscal pathway could allow the POm to instruct the cortex how to handle the incoming lemniscal information, which, overall, produces a precise qualitative assessment of the perceived stimulus in its specific context. Therefore, the level of activity in the POm could determine the cortical sensory processing regulation. POm could detect the changes in sensory activity (stimulus intensity and duration) and could adjust the gain and timing of cortical processing accordingly.

Our results unmask a new role of POm (and maybe other “higher-order nuclei”) in cortical processing and suggest a novel framework to understand thalamocortical interaction according to which POm modulates the temporal integration window of cortical sensory responses in a POm activity-dependent manner. This could be a common feature in other sensory systems.
